# Influence of β-Cyclodextrin on the Overall Antioxidant Activity and DPPH· Reaction Kinetics of Fresh Raspberry (*Rubus idaeus* L.) and Dehydrated Strawberry (*Fragaria* × *ananassa* Duch.) Extracts

**DOI:** 10.3390/plants15010152

**Published:** 2026-01-04

**Authors:** Marinela Fiţoiu (Voin), Anamaria Pop (Mateuţ), Elena Vladu, Roxana Poja, Lavinia-Alexandra Toporîşte, Carina Elena Molnar, Mărioara Drugă, Gabriel Stelian Bujancă, Ioan David, Adina Horablaga, Nicoleta-Gabriela Hădărugă, Daniel-Ioan Hădărugă

**Affiliations:** 1 Doctoral School “Engineering of Vegetable and Animal Resources”, University of Life Sciences “King Mihai I” from Timişoara, Calea Aradului 119, 300645 Timişoara, Romania; marinela.voin@gmail.com (M.F.); popdanamaria@yahoo.com (A.P.); elenablatnyak@yahoo.comroxana.poja@yahoo.com (R.P.); 2 Department of Applied Chemistry, Organic and Natural Compounds Engineering, Polytechnic University of Timişoara, Vasile Pârvan Bd. 6, 300223 Timişoara, Romania; l_t55@yahoo.com (L.-A.T.); ranlom.carina@yahoo.com (C.E.M.); 3 Department of Food Science, University of Life Sciences “King Mihai I” from Timişoara, Calea Aradului 119, 300645 Timişoara, Romania; marioaradruga@usvt.ro (M.D.); ioandavid@usvt.ro; 4 Department of Food Control and Expertise, University of Life Sciences “King Mihai I” from Timişoara, Calea Aradului 119, 300645 Timişoara, Romania; gabrielbujanca@usvt.ro; 5 Department of Sustainable Development and Environmental Engineering, University of Life Sciences “King Mihai I” from Timişoara, Calea Aradului 119, 300645 Timişoara, Romania; adinahorablaga@usvt.ro

**Keywords:** antioxidants, anthocyanin, cyanidin 3-*O*-glucoside, fruit extracts, cyclodextrin assisted dehydration, cyclodextrin assisted DPPH· method, reaction kinetics

## Abstract

The influence of natural β-cyclodextrin (β-CD) on the overall antioxidant activity of berry extracts is presented in this study. Raw raspberry (*Rubus idaeus* L.) and β-CD-assisted dehydrated strawberry (*Fragaria* × *ananassa* Duch.) ethanolic extracts (RB and SB, respectively) were spectrophotometrically monitored in the presence of 1 mM 2,2-diphenyl-1-picrylhydrazyl (DPPH·) solution in the absence or presence of β-CD. Cyanidin 3-*O*-glucoside (Cy3G) was used as standard compound, being identified by RP-HPLC in both RB and SB at 14.62 and only 0.15 mg/100 g fresh weight (fw). Pelargonidin 3-*O*-glucoside (Plg3G) was the most concentrated anthocyanin in SB (estimated at 2.46 mg/100 g fw). Higher antioxidant activities (expressed as the radical scavenging activity, RSA, %) were obtained for SB dehydrated in the presence of β-CD. The RSA values increased by 35% in comparison with the SB dehydrated by the classical method. On the other hand, the DPPH· reaction kinetic parameters significantly differed for RB extracts evaluated in the presence of 1 mM β-CD (in water). The DPPH· reaction rate in the 3–15 min time range was 25% higher for the RB extracts obtained from the β-CD-assisted dehydrated samples. This study demonstrates for the first time the protection capacity of β-CD against the degradation of antioxidants during the classical dehydration process of berries. This technology can be extended to other fruits and scaled up for obtaining high-quality fruit-based products.

## 1. Introduction

Natural antioxidants are some of the most important biologically active compounds in plants. They perform various biological activities, such as anti-aging, anti-cancer, antidiabetic, anti-inflammatory, anti-microbial, hepatoprotective, nephroprotective, neuroprotective, or anti-cardiovascular disease activities [1,2,3]. However, specific natural antioxidants have specific action mechanisms that are involved in these biological activities. They are often related to the radical scavenging activity (RSA). This is the case of the anti-aging effects of curcuminoids, anti-cancer properties of catechins, stilbenoids, and anthocyanins [1,4,5]. Among the mechanisms related to radical scavenging or inducing antioxidant enzymes, other molecular mechanisms that imply antioxidants have been demonstrated [4,6,7,8,9,10,11,12,13,14,15,16,17].

Raspberry (RB) and strawberry (SB) are widely consumed fruits, either fresh or in processed food products. They are especially reach in anthocyanin antioxidants such as cyanidin 3-*O*-glucoside (Cy3G) in fresh SB (up to 280 μg/g in *Fragaria vesca* L., wild SB, and up to 180 μg/g for *Fragaria* × *ananassa* Duch. cultivars) [18,19,20,21]. Cy3G has the highest antioxidant power (AP) among anthocyanins and anthocyanidins in SB [18]. Polyphenolic compounds in RBs belong to anthocyanins, flavonols, flavones, flavanols, flavanones, catechins, isoflavonoids, hydroxycinnamic acids, as well as ellagitannins and ellagic acid [22,23,24]. The total phenolic content (TPC) depends on various factors including the cultivar, ripening, growing parameters, or storage conditions. TPC values reach 6.45 mg/g fw, depending on the cultivar. However, the mean TPC values are generally four to five times lower [24]. Among anthocyanins, cyanidin glycosides are the most important. Total anthocyanin content (TAC) can reach 1.16 mg Cy3G/g fw [25,26,27,28].

The anthocyanin stability in processed or stored berry fruits is consistently low, especially at higher temperatures, oxidative conditions and the presence of light. The degradation of pelargonidin 3-*O*-glucoside (Plg3G) at high and low temperatures revealed half-time values up to 137 min at 100 °C and 80 days at 25 °C [18]. The total loss of anthocyanins from Bordo grape extracts is almost 89% in the liquid extract and 48% in the freeze-dried extract. The degradation of anthocyanins is significantly reduced (16.5–30%) in maltodextrin-based spray-dried samples [29]. The stability of cyanidin 3-*O*-rutinoside in black RB pomace extracts is very low, especially during the storage at 25 and 40 °C in water (residual content less than 5%). However, the stability is significantly increased for extracts obtained with natural deep eutectic solvents (NaDESs) of about 90 and 50% at 25 °C and 40 °C, respectively [30]. The stability of anthocyanins and the other antioxidants strongly depends on the processing parameters of berry fruits, including extraction, advanced non-thermal (chilled or frozen berries, preservation by pulsed light treatments, high-pressure processing, etc.), and especially thermal processing of berry fruits such as drying and dehydration, pasteurization, cooking, canning, fermentation for obtaining purée, juice, concentrate and syrup, jelly, jam, or dried berries [27,31].

Drying and dehydration are widely used for fruit preservation. Both processes remove moisture/water from fruits. Dehydration generally removes up to 70–90% moisture from the fresh fruits and, consequently, the shelf life is only few months. On the other hand, drying removes moisture down to a content inhibiting bacteria and mold development. The shelf life of the dried products is many years, such as in the case of spray-drying or freeze-drying [32,33,34,35]. Various dehydration and drying methods were used for extending the shelf life of fruits. The classical ones are air-drying, sun and solar-tunnel drying, microwave-drying, and freeze- and spray-drying. For enhancing performance and reducing degradation, combined dehydration and drying techniques, as well as pretreatments, were used (microwave vacuum-drying/microwave multi-flash drying, microwave-vacuum drying, freeze-drying combined with the far-infrared drying, blanching, osmotic pretreatment, immersing in various solutions) [32]. Convective or osmotic dehydration (ultrasound-assisted in some cases) under high hydrostatic pressures were used for the dehydration of SB [34,36,37]. Drying SB and RB involved classical convective, infrared, solar, freeze- and spray-drying, or combined techniques [38,39,40,41,42,43,44].

Stabilization and extending of the SB and RB shelf life can be performed using additives obtained by micro- and nanoencapsulation or biofilm coatings before or after processing [45,46,47,48]. Natural or modified cyclodextrins (CDs) were widely used in this regard. They are cyclic oligosaccharides containing six to eight α-d-glucopyranose units, having molecular encapsulation, controlled release, enhanced stability, and apparent water solubility properties of hydrophobic biologically active compounds. Thus, edible biofilms based on *Cryptococcus laurentii* (Kuff) Lodder for the inhibition of the mold growth, in combination with alginate, fatty acids, fatty acid monoglycerides, and β-cyclodextrin (β-CD), were used for coating the SBs, with improved quality and storage properties [49]. Chitosan/polyvinyl alcohol film loading hop β-acids/β-CD inclusion complex was used for extending the shelf life of the SBs [45]. Nerolidol/hydroxypropyl-β-CD inclusion complex nanofibers were used as ingredient for obtaining active SB packaging [48]. An antibacterial hydrogel based on κ-carrageenan, and carvacrol/hydroxypropyl-β-CD composite for SB preservation, was successfully applied [46]. A coating formulation based on whey protein, glycerol, lactose, and β-CD in post-drying and freeze-drying of SB pieces was also used [50]. RB preservation was performed by improving the performance of the konjac glucomannan film with β-CD/eugenol-based nanoemulsion [51].

The use of CDs during the processing of SB or RB such as dehydration or radical scavenging extracts have not yet been studied. This study aimed to evaluate for the first time the influence of β-CD on the convective air-dehydration of the β-CD pretreated SB (*Fragaria* × *ananassa* Duch.) slices and on the RSA of antioxidants/anthocyanins from RB (*Rubus idaeus* L.) extracts through the 2,2-diphenyl-1-picrylhydrazyl (DPPH·) kinetic parameters values. The mentioned substance was expected to modulate the overall antioxidant activity, rate constant, and half-time values during the reaction of SB and RB antioxidants with DPPH· and reveal the importance of using this natural and GRAS-approved additive for enhancing the quality of the processed berry fruits.

## 2. Results

### 2.1. Obtaining the Berry Extracts and Quantification of Anthocyanins

SB and RB extracts were obtained by semi-continuous Soxhlet extraction using well-grounded fresh fruits and ethanol as a solvent at a mass–volume ratio of 1:12. The extraction cycles were at least five. However, the multiple extraction continued until no color was observed for the last cycle. Thus, nine and five cycles were performed for obtaining the SB and RB extracts, respectively. The raw extract volumes were 297.1 ± 0.2 mL. For a proper comparison, every extract was completed with ethanol to the final volume of 300 mL.

Quantification of the main anthocyanins in SB and RB extracts was performed using the calibration curves for Cy3G and cyanidin, Cy (Equations (1) and (2) and Figures S1 and S2 in the Supplementary Materials file), which had limit of detection (LOD) and limit of quantitation (LOQ) values of 2.54 and 4.23 μg/mL for Cy3G, respectively, 1.31 and 2.18 μg/mL for Cy (see Section 4.4). The calibration curve for Cy3G was also used for the quantification of the total anthocyanin and anthocyanidin content (as mg Cy3G/100 g), considering all HPLC peaks identified at 525 nm.

Conc._Cy3G_ (mg/mL) = 0.001692(±0.000033)·Area_HPLC(Cy3G)_ n = 6, r = 0.9990, r^2^ = 0.9981, F = 2589, p < 10^−5^, s = 0.015 (1)

Conc._Cy_ (mg/mL) = 0.000651(±0.000007)·Area_HPLC(Cy)_ n = 7, r = 0.9997, r^2^ = 0.9994, F = 9246, p < 10^−5^, s = 0.008 (2)

**Figure 1:**
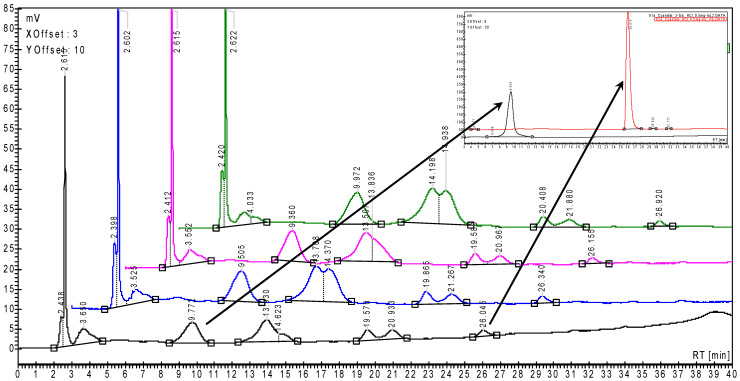
Superimposed of the representative HPLC chromatograms for the raspberry extracts (multiplicate samples). Cyanidin 3-*O*-glucoside (left arrow) and cyanidin (right arrow) were identified and quantified using the corresponding calibration curves obtained for the standard compounds by HPLC—upper right corner.

**Figure 2:**
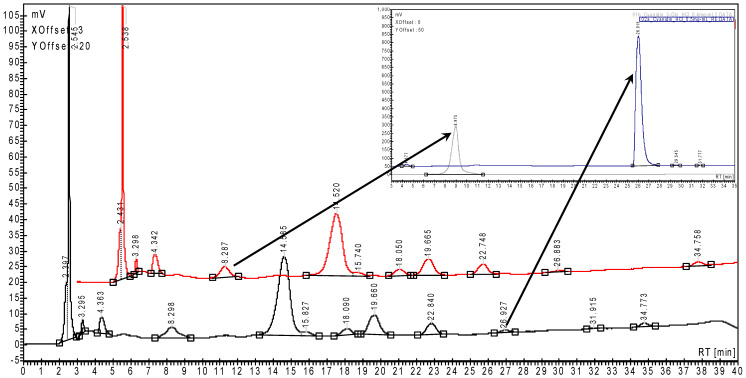
Superimposed of the representative HPLC chromatograms for the strawberry extracts (duplicate samples). Cyanidin 3-*O*-glucoside (left arrow) and cyanidin (right arrow) were identified and quantified using the corresponding calibration curves obtained for the standard compounds by HPLC—upper right corner. Pelargonidin 3-*O*-glucoside was identified (tentatively) at RT ~14.5 min according to [18,19,20].

Representative HPLC chromatograms for the fresh SB and RB extracts are presented in Figure 1 and Figure 2 (multiplicate samples). In the upper right corner of these figures are presented the HPLC chromatograms for the standard anthocyanins and anthocyanidins (Cy3G and Cy). However, the most concentrated anthocyanin in SB extracts was Plg3G, which was quantified using the same Cy3G standard curve [18,20,52]. Thus, the Cy3G content in fresh SB and RB was 14.62 ± 2.64 and only 0.32 ± 0.08 mg/100 g fw, while the total anthocyanin and anthocyanidin contents were 73.18 ± 12.39 and 4.28 ± 0.56 mg Cy3G/100 g (Table 1 and Table 2). In both extracts, the Cy content was significantly lower (0.64 ± 0.07 and 0.017 ± 0.003 mg/100 g fw, respectively).

### 2.2. Dehydration of Strawberry Slices with or Without β-Cyclodextrin as an Additive

Dehydration was only applied for SB slices. SBs are most often subjected to dehydration in comparison with other berries such as RB [42,53]. Generally, RBs are subjected to drying at significantly lower moisture content and do not fit the scope of the present study [44,54].

β-CD-assisted dehydration was applied for the first time and consisted of uniformly distributing the β-CD powder on the surface of SB slices. The retained amount of β-CD was determined as the difference between the starting amount of β-CD (excess) and the remaining amount that was not retained by the fruit slices. Approximately 1.4–1.5% of β-CD powder (related to the fresh fruit slices) was used for the dehydration process. Classical convective air-dehydration was applied.

SB dehydration reduced the mass of the fruit slices by approximately ten times. The total moisture removed from the SB slices (without β-CD) was 90.56%, while the β-CD-assisted dehydration removed 90.31% of the moisture from the fresh SB slices (see Section 4.2 for further details).

Interestingly, only the β-CD-assisted dehydrated SBs reveal significant anthocyanin content. For β-CD-assisted dehydrated SB slices, the Cy3G content was 0.15 mg/100 g and the Plg3G content was 1.64 mg (as Cy3G)/100 g. On the contrary, the cyanidin aglycone content significantly increased at 0.033 mg/100 g after dehydration at 70 °C (Table 2).

### 2.3. Antioxidant Activity of the Berry Extracts from the Raw and Dehydrated Fruits

The overall antioxidant activity of the berry extracts was evaluated using the actual RSA values during the reaction of the complex mixture of antioxidant compounds in extracts with the DPPH· radical. This free radical mimics the free radicals in the human body. These complex reactions were spectrophotometrically monitored at the maximum absorption of the DPPH· radical, i.e., 517 nm. However, some anthocyanins also absorb at this wavelength, but their absorption values at the concentration used are significantly lower than for the DPPH· radical solution (with molar absorption of ε_DPPH·_ = 1.25·10^4^ L·mol^−1^·cm^−1^ in ethanol [55,56]; see Figures S3–S8 in the Supplementary Material file). Consequently, this absorbance was subtracted from the DPPH· absorbance values during monitoring.

**Figure 3:**
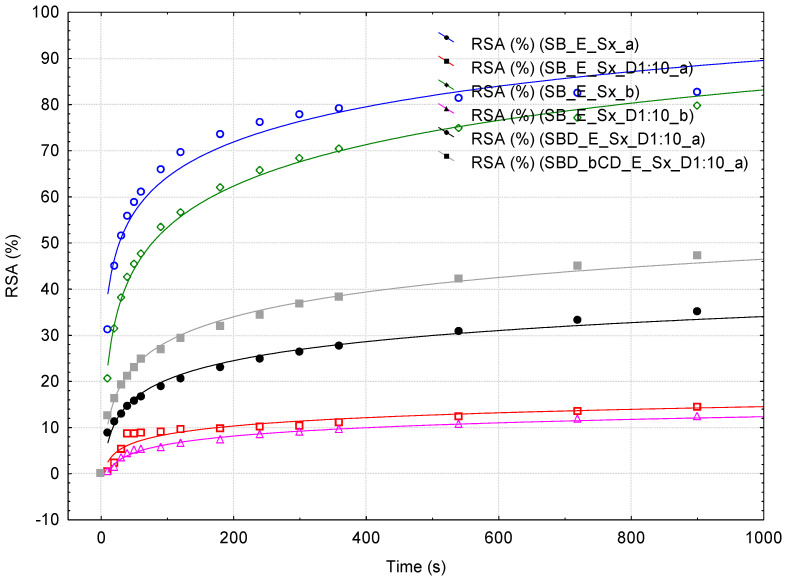
Antioxidant activity of the fresh strawberry extracts (duplicates; blue circle and green diamond), 1:10 diluted fresh strawberry extracts (duplicates; red square and pink triangle), and 1:10 diluted extracts obtained from the dehydrated strawberries, without (black circle) and with β-cyclodextrin (gray square) as an additive. Antioxidant activity is expressed as the variation in the Radical Scavenging Activity (RSA, %) in Time (s) for 15 min. Logarithmic correlations are also provided.

**Figure 4:**
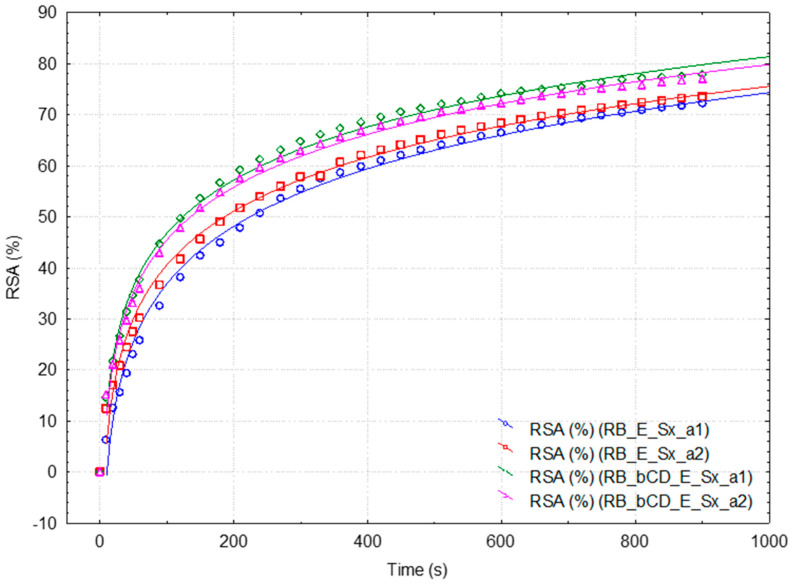
Antioxidant activity of the fresh raspberry extracts, β-cyclodextrin assisted (duplicates; green diamond and pink triangle) or not assisted (duplicates; blue circle and red square), during spectrophotometric monitoring. Antioxidant activity is expressed as the variation in the Radical Scavenging Activity (RSA, %) in Time (s) for 15 min. Logarithmic correlations are also provided.

**Figure 5:**
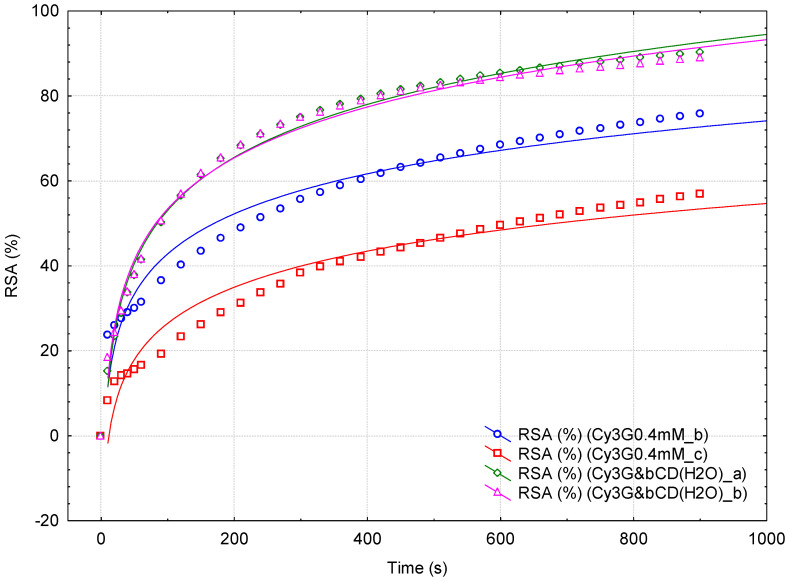
Antioxidant activity of the standard 0.4 mM cyanidin 3-*O*-glucoside, β-cyclodextrin assisted (green diamond and pink triangle) or not assisted (blue circle and red square), during spectrophotometric monitoring (duplicate samples). Antioxidant activity is expressed as the variation in the Radical Scavenging Activity (RSA, %) in Time (s) for 15 min. Logarithmic correlations are also provided.

The variation in the RSA values in time has a logarithmic behavior, especially at high dilutions (Figure 3 and Figure 4, Figures S9–S13 in the Supplementary Materials file). For comparison, similar studies were performed for the standard 0.4 mM Cy3G solutions, not assisted or β-CD assisted during spectrophotometric monitoring (Figure 5 and Figure S14 in the Supplementary Materials file). In all cases, the DPPH· reaction is very rapid in the first time range of approximately 30 s, followed by a slower reaction for the next time range (up to approximately 3 min). Then, the DPPH· reaction becomes much slower, even after the monitoring period of 15 min. Consequently, three time values were selected (i.e., ½, 3 and 15 min) for comparing the RSA values. The mean RSA values for the extracts obtained from the fresh berries were 44.84 and only 18.22% for the SB and RB extracts at ½ min, while these values significantly increased to 67.80 and 46.96, respectively, 81.17 and 72.93% for 3 and 15 min (Table 3 and Table 4). In the case of RB extracts, these values are comparable with the RSA values for the standard 0.4 mM Cy3G solution (20.92, 37.76, and 66.37% at ½, 3 and 15 min, respectively, Table 5).

The extracts obtained from the dehydrated berries have significantly higher RSA values, especially for the β-CD-assisted samples at ½ and 3 min (between 81.47 and 84.32%, Table 3). The antioxidant activity of the raw extracts of the dehydrated SBs was significant even at the beginning of the spectrophotometric monitoring. Consequently, the 1:10 dilution of the extracts were analyzed in the same way. The RSA values decreased at 4.37% at ½ min for the SB diluted extract, while the corresponding values for extracts obtained from the β-CD-assisted or not-assisted dehydrated samples were significantly higher (19.19 and 13.04%, p < 0.01). At the end of the spectrophotometric monitoring, the differences were also significant (47.32% for the case of β-CD-assisted dehydrated SB, 35.14% for the not-assisted dehydrated SB and only 13.40% for the diluted extract obtained from the fresh SB, p < 0.01, Table 3).

The influence of β-CD on the overall antioxidant activity was observed during spectrophotometric monitoring. The RSA values are 43.5, 18.5, and 6.1% higher for fresh RB extracts with β-CD-assisted spectrophotometric monitoring at ½, 3 and 15 min, respectively (Table 4). This behavior was also observed for the standard 0.4 mM Cy3G solution. The RSA values were 35% higher for the RB extract analyzed in the presence of β-CD in comparison with the not-assisted samples (Table 5).

### 2.4. Kinetics of the DPPH· Reaction with Antioxidant Compounds in Berry Extracts with or Without β-Cyclodextrin as an Additive

DPPH· reaction kinetics can provide information on the overall differences between β-CD-assisted or not-assisted dehydration, respectively, and spectrophotometric monitoring (β-CD can modulates the DPPH· reaction with various antioxidant compounds). Both DPPH· reaction constant, k_n_, and half-time, t_1/2_, are important in this regard. Due to the very complex reactions of DPPH· radical with antioxidant compounds in extracts (anthocyanins, anthocyanidins, flavonoids, hydroxycinnamic acids, or ellagitannins), with higher concentrations compared to DPPH·, the kinetic models were only related to DPPH· [57,58]. The DPPH· also reacts with various species in the case of Cy3G (more reaction sites on the Cy3G, as well as further reaction with the products having antioxidant activity) [59,60].

All kinetic results are presented in the Supplementary Materials file. The linear correlation of the integrated reaction rate law on the selected time ranges of 0–30 s and 30–180 s (“fast” and “slow” reactions [61]; Figures S15–S20 in the Supplementary Materials file) allows determining the reaction rate constants and half-times for the zeroth- to fourth-(pseudo-)order kinetic models (Tables S1–S6 in the Supplementary Materials file; tables also contain the determination coefficients, r^2^, for the integrated reaction rate law correlations). Generally, the best results for the berry extracts were obtained for the higher-(pseudo-)order kinetic models (third- and fourth-order kinetic models). On the contrary, better statistical parameters for the standard antioxidant compound Cy3G were obtained for the first- and second-(pseudo-)order kinetic models.

**Figure 6:**
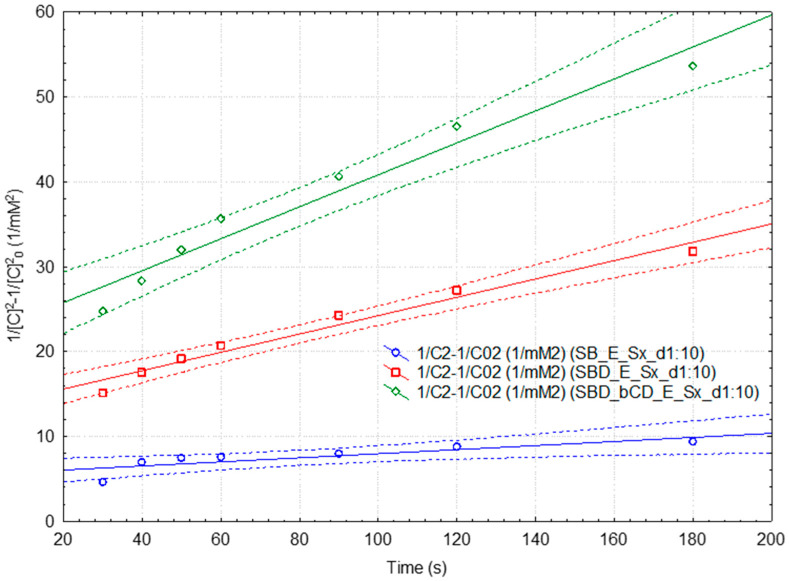
Integrated rate law representation for the third (pseudo-)order kinetic model for the “slow” DPPH· reaction with antioxidant compounds from the extracts obtained from the fresh and dehydrated strawberries (without or with β-cyclodextrin as additive; 1:10 diluted extracts). Mean values and 95% confidence interval (dotted lines) were used.

Representative third-(pseudo-)order kinetic models for the “fast” and “slow” DPPH· reaction with antioxidant compounds in extracts obtained from the fresh and dehydrated SB (without or with β-CD as additive) are presented in Figure 6 and Equations (3)–(5). All kinetic data for these samples are presented in the Figures S15 and S16, respectively; Tables S1 and S2 from the Supplementary Materials file. For the third-(pseudo-)order kinetic model, both dehydrated samples reveal higher rate constant of 0.794 and 0.485 mM^−2^·s^−1^, three to five times higher than the corresponding rate constant for the fresh SB extract in the case of “fast” reaction. On the other hand, the half-time values are in the range of 1.4–7.6 min, significantly lower for the β-CD-assisted dehydrated SB samples (p < 0.05). These aspects can also be observed for the “slow” reaction. However, better statistical parameters were obtained for the third-(pseudo-)order kinetic models (r^2^ in the range of 0.96–0.97 for the dehydrated SB samples, Equation (5)). The rate constant and half-time values for the dehydrated samples are in the range of 0.108–0.188 mM^−2^·s^−1^ and 6.1–10.6 min, significantly different from the fresh SB samples case (k_3_ = 0.024 mM^−2^·s^−1^ and t_1/2(3)_ = 47.6 min, p < 0.001 and p < 0.01, respectively).

**Figure 7:**
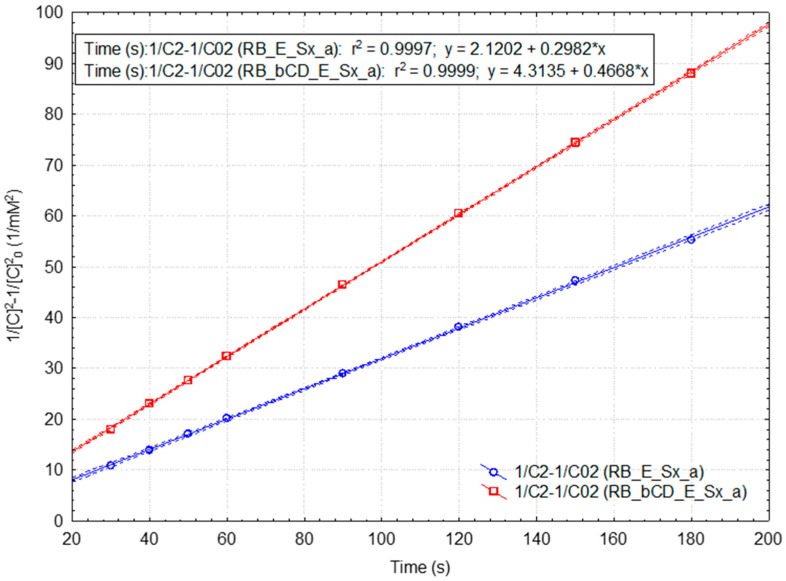
Integrated rate law representation for the third (pseudo-)order kinetic model for the “slow” DPPH· reaction with antioxidant compounds from the extracts obtained from the fresh raspberries (without or with β-cyclodextrin as additive during the spectrophotometric monitoring). Mean values and 95% confidence interval (dotted lines) were used.

**Figure 8:**
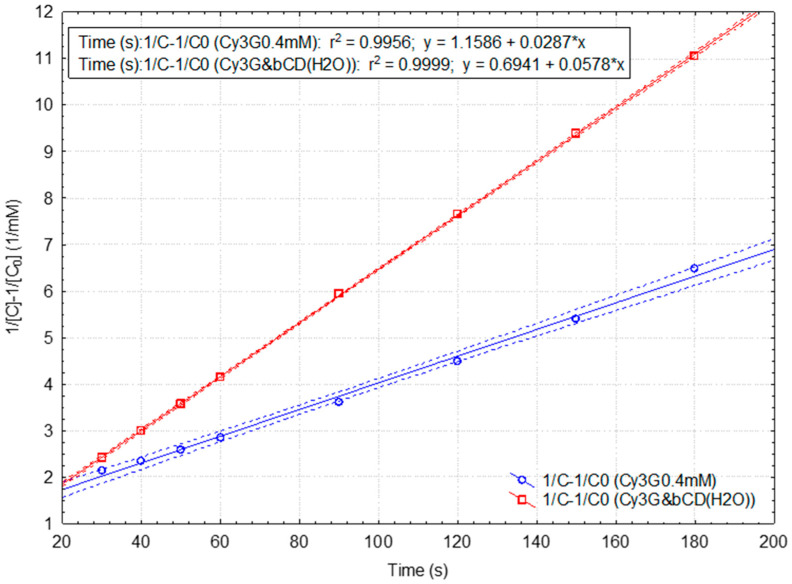
Integrated rate law representation for the second (pseudo-)order kinetic model for the “slow” DPPH· reaction with the standard 0.4 mM cyanidin 3-*O*-glucoside (without or with β-cyclodextrin as additive during the spectrophotometric monitoring). Mean values and 95% confidence interval (dotted lines) were used.

Kinetic parameters were significantly enhanced for the RB samples, especially for the β-CD-assisted samples during spectrophotometric monitoring (Figures S17 and S18, respectively, Tables S3 and S4 in the Supplementary Materials file). For the “fast” reaction, the rate constant and half-time values of the β-CD-assisted and not-assisted RB samples were 0.590 and 0.358 mM^−2^·s^−1^, respectively, 54.5 and 89.8 s. For the “slow” reaction, the corresponding kinetic parameters were different, but not such as in the case of SB samples. On the other hand, the statistical parameters for the kinetic models are significantly enhanced (r^2^ = 0.99994 for the third-(pseudo-)order “slow” reaction model in the case of the β-CD-assisted RB sample; Figure 7 and Equations (6) and (7)). The statistical parameters for the case of the “slow” reaction of DPPH· with the standard 0.4 mM Cy3G solution in the presence of β-CD are similar, but for a lower reaction order (r^2^ = 0.99991 for the second-(pseudo-)order reaction kinetics; Figure 8 and Equations (8)–(10)). The rate constant is two times higher for the β-CD-assisted RB sample, while the half-time value is 50% from the value corresponding to the not-assisted RB case (1.7 and 3.4 min, respectively). All results for the kinetic studies related to the reaction of DPPH· with the standard Cy3G solutions, β-CD-assisted or not-assisted during spectrophotometric monitoring, are presented in Figures S19 and S20, respectively; Tables S5 and S6 from the Supplementary Materials file.

(3) $$\frac{1}{{\left[C\right]}^{2}}-\frac{1}{{\left[C\right]}_{0}^{2}}=2\cdot k_{3}\cdot t;\;t_{1/2(3)}=\frac{3}{2}\cdot \frac{1}{k_{3}\cdot {\left[C\right]}_{0}^{2}}$$

(4) $$y=2.86+0.794\left(\pm 0.165\right)\cdot x\left(“fast”\;reaction\;for\;the\;\beta \text{-}CD\;dehydrated\;SB\;extract\right)\;\;r=0.959,r^{2}=0.920,F=23.1,p<0.05,s=3.69$$

(5) $$y=22.00+0.188\left(\pm 0.018\right)\cdot x\left(“slow”\;reaction\;for\;the\;\beta \text{-}CD\;dehydrated\;SB\;extract\right)\;\;r=0.978,r^{2}=0.957,F=111.0,p<0.0002,s=2.34$$

(6) $$y=1.019+0.590\left(\pm 0.059\right)\cdot x\left(“fast”\;reaction\;for\;the\;\beta \text{-}CD\text{-}assisted\;RB\;extract\;case\right)\;\;r=0.990,r^{2}=0.980,F=99.8,p<0.01,s=1.32$$

(7) $$y=4.314+0.467\left(\pm 0.001\right)\cdot x\left(“slow”\;reaction\;for\;the\;\beta \text{-}CD\text{-}assisted\;RB\;extract\;case\right)\;\;r=0.99997,r^{2}=0.99994,F=1019,p<0.00001,s=0.21$$

(8) $$\frac{1}{\left[C\right]}-\frac{1}{{\left[C\right]}_{0}}=k_{2}\cdot t;\;t_{1/2(3)}=\frac{1}{k_{2}\cdot {\left[C\right]}_{0}}$$

(9) $$y=0.587+0.061\left(\pm 0.002\right)\cdot x\left(“fast”\;reaction\;for\;the\;\beta \text{-}CD\text{-}assisted\;Cy3G\;solution\right)\;\;r=0.999,r^{2}=0.998,F=642.2,p<0.03,s=0.03$$

(10) $$y=0.694+0.058\left(\pm 0.000\right)\cdot x\left(“slow”\;reaction\;for\;the\beta \text{-}CD\text{-}assisted\;Cy3G\;solution\right)\;\;r=0.99996,r^{2}=0.99991,F=69712,p<0.00001,s=0.03$$

## 3. Discussion

This study focuses on the influence of natural β-CD during both convective air-dehydration of berries and reaction of antioxidants in berry extracts with free radicals, which has been investigated and is reported here for the first time. There were statistically significant differences in terms of antioxidant activity and radical reaction kinetics.

### 3.1. Identification and Quantification of Anthocyanins

Eleven anthocyanins and anthocyanidins were separated using HPLC in SB extracts. The identification and quantification was performed for the main antioxidant compounds in SB, i.e., Cy3G and Cy. Plg3G and total anthocyanin and anthocyanidin content were also estimated as Cy3G. Cy3G content in fresh SB was 0.32 ± 0.08 mg/100 g fw, but Cy was identified at only 0.017 ± 0.003 mg/100 g fw. However, Plg3G was the most concentrated in fresh SB, being identified at 2.46 ± 0.38 mg/100 g fw, more than a half of the total anthocyanin and anthocyanidin content (4.28 ± 0.56 mg Cy3G/100 g fw). The identification of the main anthocyanins in berry extracts was also supported by the statistically significant correlation of the retention time (RT) parameter in RP-HPLC analysis with the logarithm of the octanol/water partition coefficient, LogP, which is strongly related with the partition of the antioxidant compounds between the hydrophobic stationary phase (Nucleosil 100 C18 column based on octadecylsilane structure) and the hydrophilic mobile phase (acidified acetonitrile–water mixture) [62,63]. LogP values for anthocyanins were calculated using the atomic contributions implemented in the QSAR Properties module in HyperChem 7.52 (HyperCube, Inc., Gainesville, FL, USA). Further information can be found in the Figures S21–S23 in the Supplementary Materials file. Thus, for the identified anthocyanins, i.e., Cy3G, Plg3G, and Cy, the RT versus logP correlation is given below (Equation (11)).

(11) $${RT}_{HPLC}\left(min\right)=10.444\left(\pm 0.564\right)\cdot LogP\;r=0.997,r^{2}=0.994,F=342.4,p<0.003,s=1.58$$

SB composition is in line with other research on the fresh berries. However, there are large differences in both the specific anthocyanin content and total anthocyanin content. De-Souza and collaborators determined the total anthocyanin content at 16.03 mg Cy3G/100 g fw [25], while Chaves et al. quantified the total anthocyanins in fresh SB at 15.67–23.46 mg Plg3G/100 g [19]. Similar values of 15.45 and 19.95 mg/100 g fw were obtained after various pretreatments of the plants (e.g., Lemon verbena bio extract applied to SB plants at 200–600 mg/L) [64,65]. Higher total anthocyanin contents of 38–176 mg/100 g and 98 mg Cy3G/100 g fw were obtained by Dzhanfezova et al. and Jiang et al. [20,42]. On the other hand, Hussain et al. determined lower content of anthocyanins in fresh SB at only 3.17 mg/100 g fw [3]. Plg3G was determined in fresh SB at the highest content (up to 170 mg/100 g fw) by comparison with the other anthocyanins. However, the Plg3G content was quantified at 0.31–59.86 [21], 4.157–6.235 [18], 14.05–22.30 [19], 18.02 [65], 25.066 [52], and 30–170 mg/100 g [20]. In all cases, Cy3G was less concentrated, with a range of 0.68–28 mg/100 g in fresh SB (0.68 [52], 0.8–28 [20], 0.83–1.99 [19], 1.39 [65], 1.988–2.774 [18], and up to 6.01 mg/100 g [21]).

Fresh RB had higher total anthocyanin and anthocyanidin content of 73.18 ± 12.39 mg Cy3G/100 g fw, while the specific Cy3G and Cy were identified at 14.62 ± 2.64 and 0.64 ± 0.07 mg/100 g fw, respectively. These values are in agreement with the other results obtained by Wang et al. for RB of 6.39 ± 0.02 mg Cy3G/100 g fw, among other anthocyanins such as cyanidin 3-sophoroside or cyanidin 3-(6′-citryl)-sophoroside [28]. However, the total anthocyanin content determined in other studies vary in a wide range of 14.69–98.91 mg/100 g, depending on the RB growing and processing conditions [25,26,28]. For example, these values increased after using biostimulants at 10.71–24.85 mg Plg3G/100 g in the first year and to 35.12–58.87 mg Plg3G/100 g for the second year of treatment [26]. On the other hand, the total anthocyanin content of the RB subjected to subcritical water extraction at 7 MPa and 130 °C for 90 min reached 98.91 ± 0.33 mg/100 g [28].

### 3.2. Dehydration of Strawberry Fruits

The dehydration and drying of fruits induces the partial degradation of antioxidant compounds, especially if the air–oxygen and higher temperature are used like in the case of classical convective air-dehydration used in this study, which reduced the moisture content of SB by 91%. The total anthocyanin content, as well as the content of the main anthocyanins in β-CD-assisted dehydrated SB was slightly higher than in the fresh SB (4.30 mg Cy3G/100 g for the total anthocyanin and anthocyanidin content). On the other hand, the anthocyanin compounds were identified at lower contents, but their aglycones were more concentrated in the β-CD-assisted dehydrated SB. For example, only 1.64 and 0.15 mg/100 g were determined for Plg3G and Cy3G, but the aglycone Cy was more concentrated (0.033 mg/100 g). Unfortunately, the dehydration without β-CD addition provided products with very low content of anthocyanins and anthocyanidins, which most probably were degraded to other compounds during the longer thermal process. There are various studies on the degradation of anthocyanins as pure compounds or in fruit extracts. Cy3G content is reduced at a third from the starting concentration in ethanol solution subjected to 95 °C for 30 min, especially under pressure [66]. The PEF (Pulsed Electric Field) processing also affect the stability of Cy3G [67]. Anthocyanins in blueberry extracts were significantly degraded under heat and acid treatments. Almost no Cy3G, as well as malvidin 3-*O*-glucoside, peonidin 3-*O*-glucoside, malvidin 3-*O*-galactoside, petunidin 3-*O*-glucoside, petunidin 3-*O*-galactoside, delphinidin 3-*O*-glucoside, cyanidin 3-*O*-galactoside, delphinidin 3-*O*-galactoside, or cyanidin 3-*O*-arabinoside, were identified after the treatment of TFA (trifluoroacetic acid) acidified blueberry extract at 95 °C for 90 min [68]. The anthocyanin content decreased very fast in SB concentrates subjected to heating at 95 °C and pH 3.5 for 6 h. The total anthocyanin content decreased at only 40.39 mg/L from a starting value of 171.35 mg/L. The half-time for SB was the highest (1.95 h) in comparison with elderberry and black carrot concentrate cases [69]. Total anthocyanin content was slightly modified after spray-drying of SB at 8.04 mg/100 g (from the starting content of 3.17 mg/100 g in fresh SB) [3]. The total anthocyanin content was not significantly modified after various pretreatments (freeze–thaw, ultrasound, freeze–ultrasound) in infrared combined hot-air-impingement drying of SB slices, from the 98 to 108–119 mg Cy3G/100 g, related to the dry basis [42].

Many anthocyanin degradation compounds are generated under heat and oxygen, although some of them can retain the antioxidant properties. The first step is deglycosylation (hydrolysis) to the corresponding aglycone (e.g., Cy), followed by various cleavages providing smaller compounds such as phloroglucinaldehyde or protocatechuic acid [67,69]. The content of the degradation antioxidant compounds significantly increased to 11.89 μg/mL for 4-hydroxybenzoic acid and 4.89 μg/mL for phloroglucinaldehyde in the SB concentrate subjected to 95 °C and pH 3.5 for 4 h. These degradation compounds were not identified in the fresh products [69].

### 3.3. Antioxidant Activity of Berry Extracts

Antioxidant activity is one of the most important properties of berries, achieved through the radical scavenging capacity of the contained polyphenols: anthocyanins, anthocyanidins, flavonoids, hydroxycinnamic acids, and ellagitannins. However, these compounds are less stable, especially during the thermal processing or in the presence of air [22,23,70,71]. Consequently, the use of β-CD as natural additive for convective air-dehydration of berry slices, as well as during the radical scavenging reactions of antioxidant compounds contained by berry extracts, was focused on the protection (enhancing stability) of antioxidant compounds and the modulation of their reactions with free radicals [72,73]. These applications of β-CD were studied for the first time and the results are very promising.

In all studied cases, β-CD addition enhanced the overall antioxidant activity, as revealed by the actual and final RSA values. This behavior was easily observed for the diluted extracts obtained from the β-CD-assisted dehydrated SB, by comparison with the not-assisted samples or the raw RB extracts. The RSA values were 3.5 times higher for the β-CD-assisted dehydrated samples and these differences are slightly lower for the β-CD-assisted reaction of free radicals with the antioxidant compounds in RB extracts, but statistically significant (p < 0.05). At the final monitoring, the RSA values for the β-CD-assisted reaction were 6% higher in comparison with the not-assisted reaction. However, the low solubility of β-CD in ethanol determined the use of ethanol–water solution at high dilution for maintaining the complete solubilization of both β-CD and its complexes with antioxidant compounds from the various classes, reaction products, and even free radicals.

The enhancement of the antioxidant activity by the β-CD is due to the efficient interaction of the more hydrophobic and geometrically compatible antioxidant moiety with the hydrophobic cavity of β-CD. Further, the hydrophilic moieties that contain phenolic groups are exposed to the solution (including by retention of these OH groups at the surface of the complex through H-bonding). These orientations of the antioxidant compounds enhanced the interaction with the free radicals, which mainly remains in the solution. DPPH· has a relatively rigid and bulk-like structure that is not compatible with the β-CD cavity (see Figures S24 and S25 in the Supplementary Materials file). This can also explain the differences between the RSA values, which are higher for the standard Cy3G solution analyzed in the presence of β-CD, in comparison with the not-assisted sample case. Moreover, the stabilization of the antioxidant compound during analysis can provide better reproducibility for this reaction. Significantly lower SD values were obtained for β-CD-assisted spectrophotometric reaction monitoring. However, further studies are needed for proving this observation.

The antioxidant activity of SB and RB extracts was determined in various studies for fresh or processed fruits. The most used method was the DPPH· assay, but ABTS^+^· (2,2′-azino-bis(3-ethylbenzothiazoline-6-sulfonic acid) radical cation), FRAP (ferric reducing antioxidant power), or TEAC (trolox equivalent antioxidant capacity) were also applied. Up to now, no studies regarding the modulation of the antioxidant activity by CDs were reported. However, the overall antioxidant activity of SB and RB varies in wide ranges, but the processing (thermal and/or oxidative) significantly decreases the antioxidant activity related parameter values, as was discussed in the present study for the dehydration process. Relevant published studies on antioxidant activity of both fresh and processed berries are discussed below.

Fresh SB and RB were studied for their antioxidant activity using DPPH·, ABTS^+^·, and β-carotene assays. The results revealed values of 3779 and 4961 g fw/g DPPH·, 7.87 and 6.27 μmol TE/g fw, respectively, and 67.13 and 75.19% protection [25]. DPPH· and FRAP assays were also applied for 14 SB cultivars that reveal high antioxidant activity during the two years of monitoring. The highest values of 9.1–11 mmol TE/g fw, respectively, 45–50 mmol Fe^2+^/g fw, were obtained for wild SB (*Fragaria vesca* L.). By comparison, DPPH· and FRAP assays reveal values in the ranges of 3.1–5.6 mmol TE/g fw and 13.8–30 mmol Fe^2+^/g fw for the *Fragaria* × *ananassa* Duch. cultivars [20]. Mandrave et al. determined the antioxidant activity of other two SB “Sweet Charlie” and “Camarosa” cultivars at four different fruit developmental stages. EC_50_ values for the DPPH· and FRAP assays were in the ranges of 9.71–130.4 mg/mL and 57.11–219.5 μg for the 0.2% acetic acid extracts, respectively, and 39.01–296.85 mg/mL and 13.90–202.03 μg for the ethanol extracts [74]. In another study on the 90 SB (*Fragaria* × *ananassa* Duch.) cultivars, the DPPH· and ABTS^+^· assays provide values of 751.57–765.06 and 1531.17–1637.96 μmol TE/100 g fw for two years of monitoring [21]. Applying nano-fertilizers during the growing of berry plants influences the antioxidant activity of the berries, as was determined by the DPPH· scavenging activity for SB. The best result of 93.88% was obtained for a combined application into soil of a hybrid nanocomposite and a nano-zeolite fertilizer [75].

A study on the variation in the antioxidant activity (determined by TEAC assay) during heating of SB pigment solutions at 95 °C and pH 3.5 for 4 h revealed decreases in the TEAC values from 185.38 to 138.00 mg vitamin C equivalent/L, which means a reduced antioxidant activity by 25% [69]. On the other hand, high-intensity ultrasound treatment of the SB juice increased the antioxidant activity by 38%, as was determined using the DPPH· method. The DPPH· inhibition value increased from 31.5% to 43.5% for the ultrasound-treated samples (20 kHz, 400 W, and 12 min) [76].

Antioxidant activity of fresh red and black RB at various development stages was evaluated using FRAP, ascorbic acid (ASA), and GSH assays. Generally, the red RB had a slight increase or not-significant antioxidant activity during the developing stages. For red RBs, there were four stages of development, starting from the small immature green fruits to fully ripe dark red fruits easily detached from the crown. On the other hand, for black RBs, there were five stages of development from the small immature green fruits to fully ripe purple fruits. The black RB had a significant increase in the antioxidant activity parameters values at the fifth stage of maturity (FRAP 12 μmol/g, ASA 16 mg/g fw, and GSH 12.4 mg/g fw) [77]. For another seven RB cultivars, the antioxidant activity was determined by DPPH· and ORAC assays with values of 305–350 and 136.7–205.2 μmol TE/g dw (dry weight), respectively [78]. The use of microbial biostimulants (*Bacillus subtilis* (Ehrenberg) Cohn and/or *Paenibacillus* Ash et al., 1994 species) increased the overall antioxidant activity of RB by 16–20%. For example, the DPPH· value increases from 55.02% to 62.4% for “Delniwa” cultivar if a biostimulant based on 6.25 mL liquid preparation containing *B. subtilis* (B4/19-AF75AB2) and *B. subtilis* (B7/19-Sp115AD), 6.25 mL liquid preparation containing *B. subtilis* (B7/19-Sp115AD) and *Paenibacillus* sp. (B13/19-Sp116AC), 37.5 mL water and 10 g powder preparation containing *B. subtilis* (B4/19-AF75AB2) and *Bacillus* sp. (B6/19-AF75BC) was applied on soil 2–3 times during the growing season [26]. In another study, the anthocyanins separated from RB by subcritical water extraction (at 100 μg/mL) had the highest antioxidant activity (83.77% by DPPH· assay and 66.7% by ABTS^+^· assay) in comparison with the hot water or methanol extracts at the same anthocyanin content [28].

Dehydration and drying significantly influenced the antioxidant activity of berries, which strongly depend on the technique used. Osmotic dehydration pretreatment, followed by freeze-drying of SB, decreased the antioxidant activity by DPPH· technique from 85.24% for the fresh berries to only 42.94% for the freeze-dried samples, which is in line with our results on convective air-dehydration [79]. On the other hand, infrared combined hot air impingement drying of SB slices provide antioxidant activities in the range of 62.34–62.69% if the freeze–thaw, ultrasound, or freeze–ultrasound pretreatments were used [42]. Santos et al. also used ultrasound and ethanol pretreatments for enhancing the antioxidant activity of SB slices subjected to convective air-drying. The best results were obtained for ultrasound and 50% ethanol pretreatment. The DPPH·, ABTS^+^·, and FRAP values decreases by 10.32, 13.78, and 6.54%, respectively [80].

CDs were only used for obtaining biofilms or other materials designed for preservation of fresh berries. Antioxidant activity of such preserved fruits were determined using the methods presented above. The DPPH· assay revealed values of ~48% for SB preserved with a biofilm based on chitosan/polyvinyl alcohol loading β-acids/β-CD inclusion complex in comparison with the control sample of 40% after three days of storage [45]. Enhancement of the antioxidant activity and stability was also obtained if a hydrogel based on κ-carrageenan and carvacrol/hydroxypropyl-β-CD composite, nerolidol/hydroxypropyl-β-CD inclusion complex nanofibers, or a film composite based on Pickering nanoemulsion loaded with eugenol/β-CD and konjac glucomannan were applied to SB fruits [46,48,51].

### 3.4. Kinetic Studies

Both the DPPH· and ABTS+· methods were widely used for the evaluation of the antioxidant activity of berry extracts. One of the scope of this study was the evaluation of the reaction of a free radical with antioxidant compounds in berry extracts in the absence or presence of β-CD. In this regard, the reaction kinetics (“fast” and “slow”) were evaluated. By far, DPPH· reaction kinetics is the most studied, especially for the reaction with antioxidant compounds [55,58,59,60,81,82,83,84,85]. The DPPH_2_ with absorbance < 450 nm was obtained as the reaction product. On the other hand, reaction kinetics involving ABTS^+^· radical cation is less studied. Only a few studies regarding the reaction kinetics between ABTS^+^· and flavonoids or other antioxidants were found in the literature [86,87,88,89]. Another problem is related to the formation of two species, i.e., ABTS^+^ and ABTS^2+^, as the products of the reaction with antioxidant compounds [86].

DPPH· kinetics during the reaction with antioxidant compounds in berry extracts in the presence of β-CD can provide further information on the stability and behavior of anthocyanins as free radical scavengers. β-CD comes from the assisted dehydration of SB, when β-CD is partially transferred during the extraction process or is used as additive during the DPPH· reaction with antioxidants from RB extracts. As previously presented for both “fast” and “slow” reactions (classification of the reaction types according to [61]), the best fits and statistical parameters were obtained for the higher-(pseudo-)order kinetic models (only related to DPPH· concentration), i.e., third- and fourth-(pseudo-)order kinetic models. On the other hand, pure Cy3G reveals lower-(pseudo-)order kinetic models. These differences on the kinetic behavior can be explained by the very complex antioxidant system/mixture, not only as specific polyphenols, but also as different classes of compounds. However, the DPPH· reaction behavior is similar for berry extracts and pure Cy3G assisted or not by β-CD. This is due to the favorable interactions of antioxidant compounds with β-CD cavity, regardless the chemical class of antioxidants [72,90,91,92,93,94,95,96,97,98,99,100,101]. According to these DPPH· kinetic models, the rate constant for the “fast” reaction with antioxidants from β-CD-assisted dehydrated SB was three to five times higher than the corresponding rate constant for the fresh SB extract. This behavior was also observed for the “slow” reaction, but the differences between rate constant values are even higher for the case of third-(pseudo-)order kinetic model. Consequently, the DPPH· reaction in the presence of β-CD is faster. These aspects were also observed during the DPPH· reaction with antioxidant compounds in RB extracts in the presence of β-CD, but the differences are less significant, such as for the pure Cy3G case. However, no kinetic studies were published for the DPPH· reaction with antioxidant compounds in the presence of CDs. There are some kinetic studies on the DPPH· reaction with flavonols, flavanols and hydroxycinnamic acids (rate constants for the first-order kinetics of 400–5000 L·mol^−1^·s^−1^) [59], flavonol quercetin (first-order kinetics) [60], flavanone naringenin in micellar media [85], or curcumin (rate constants of the first-order kinetics of 1.8·10^4^ L·mol^−1^·s^−1^) [82]. Other kinetic studies are related to the anthocyanin degradation processes or hydrolysis reactions under various conditions, even as pure antioxidant compounds or in fruit extracts [68,102,103,104,105,106].

All the mentioned examples focus on bimolecular reactions and were proven to follow lower-order kinetic models, such as in our study on the reaction of DPPH· with pure Cy3G. However, DPPH· kinetic models for complex reactions with antioxidants in fruit extracts are more difficult to evaluate. This is the case of “fast” and “slow” kinetics with antioxidants in citrus juices [58]. Other DPPH· kinetic studies were performed on the “extremely fast” reaction region (e.g., up to 10 s), as well as the “fast” and “slow”/”slower” regions, for gallic acid, methyl gallate, ascorbic acid, catechin, epicatechin, quercetin, rutin, tannic, ellagic, and syringic acids, rutin in micellar nanostructures, as well as for antioxidants from various fruit juices such as red plum or SB [55,61,81].

## 4. Materials and Methods

### 4.1. Plant Materials and Chemicals

Fresh SBs (*Fragaria* × *ananassa* Duch. var. Albion) were purchased from the local producer (batch number 1905, IamaAgri Strawberry Factory, Arad, Arad county, Romania; growing location: Peciu Nou, Timiş county, Romania; growing coordinates and conditions: 45°36′23″ N and 21°3′28″ E, natural pollination and biological pest control; harvesting period: May 2022). Fresh fruits were stored at 4 °C in the original package for maximum two days until the convective drying experiments.

Fresh European RBs (*Rubus idaeus* L.) were purchased as frozen whole fruits from a producer from Transylvania (batch number RO.10.VI.22, S.C. Transylvania Royal Food S.R.L., Târnăveni, Mureş county, Romania; growing coordinates: 46°19′47″ N and 24°16′12″ E; harvesting period: June 2022). The fruits were stored at −18 °C until extraction process.

Ethanol (96%, *v*/*v*, ChemiCal Co, Bucharest, Romania) was used for both extraction and spectrophotometric analysis. β-Cyclodextrin hydrate, β-CD (Kleptose^®^), was purchased from Roquette Freres S.A. (Lestrem, France) and had a purity > 98% (max. 0.25% α-CD, max. 0.25% γ-CD, max. 0.5% other impurities, max. 14% water content). Cyanidin 3-*O*-glucoside chloride, Cy3G (from *Rubus fruticosus*, purity > 97% by HPLC) and cyanidin chloride, Cy (from *Aronia melanocarpa*, purity > 97%), were purchased from PhytoPlan^®^ Diehm & Neuberger GmbH (Heidelberg, Germany). Acetonitrile (HPLC grade, Honeywell|Riedel-de Haën, Seelze, Germany) and trifluoroacetic acid (“for analysis” grade, Carlo Erba Reagents S.A.S., Val-de-Reuil, France) were used for chromatographic analysis. 2,2-Diphenyl-1-picrylhydrazyl (DPPH·) for radical scavenging activity and reaction kinetics had a purity > 99.5% and was obtained from Sigma-Aldrich Chemie GmbH (Taufkirchen, Germany).

### 4.2. Dehydration of Fruits in the Presence or Absence of β-Cyclodextrin

Dehydration was applied for fresh SB slices using a SilverCrest SDA 350 A2 (350 W) convective air-dehydrator (HOYER Handel GmbH, Hamburg, Germany). Fresh fruits were washed with distilled water, blotted with filter paper for removing the remaining water, and sliced along the longitudinal axis at a thickness of ~3–4 mm. Dehydration was performed using two methods.

**Figure 9:**
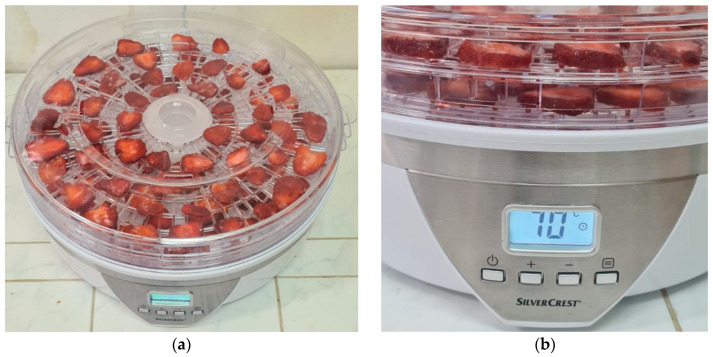
Strawberry dehydration process. (**a**) Overview of the convective air-dehydrator; (**b**) parameter setting (e.g., temperature) for the strawberry dehydration.

Classical convective air-dehydration. Around 133.6 ± 1.6 g of sliced fruits were weighted at the starting of dehydration, placed on the dehydration trays at distances of about 1 cm between slices (Figure 9). The dehydration time and temperature were set at 12 h and 70 °C, respectively (according to the producer for these types of samples). After the dehydration process, the samples were weighted again and stored at 4 °C in a sealed glass flask until extraction.

β-CD-assisted convective air-dehydration. β-CD-assisted convective air-dehydration was applied for the first time using β-CD as natural additive for enhancing the water diffusion from the inner parts of the samples as well as for protection of the antioxidant compounds against degradation (in the presence of air/oxygen and high temperature). The same amount of sliced strawberries (133.7 g) was uniformly covered with powdery β-CD hydrate (1.88 g) and immediately subjected to air-dehydration in the same conditions (70 °C for 12 h). After weighting, the dehydrated samples were stored at 4 °C until extraction.

### 4.3. Extraction of Antioxidants from Fresh and Dehydrated Fruits

Fresh and dehydrated fruits were subjected to the classical semi-continuous solid–liquid extraction according to the literature for the separation of anthocyanins and other polyphenols from fruits using ethanol as “green” solvent [107,108]. Slight modifications were applied to these methods from the literature. Thus, 250 mL or 100 mL Soxhlet extractors were used for the fresh and dehydrated samples, respectively. Soxhlet extractors were equipped with reflux condensers. Approximately 25 g of fresh fruits or 12–13 g of dehydrated fruits were finely grounded in a mortar and transferred into the extractor’s cellulose cartridge. In total, 300 mL of 96% ethanol was added to the 500 mL round-bottom flask for the fresh samples. In total, 150 mL of 96% ethanol was used for dehydrated samples. The flasks were heated on water baths and the samples were extracted for at least five times (until no color was observed in the final extract of the multiple extraction process and the HPLC analysis of the last extract did not reveal any significant peaks for anthocyanins). After cooling, all the final extracts were completed with ethanol at a final volume of 300 and 150 mL for the fresh and dehydrated samples, respectively. SB and RB extracts were hermetically sealed in glass flasks until the UV-Vis spectrophotometry and HPLC analyses (they were stored at 4 °C up to two days if necessary).

### 4.4. HPLC Analysis of the Main Anthocyanins from Fruits

HPLC analysis was performed for identification and quantification of the main anthocyanin derivatives in both SB and RB extracts—Cy3G, as well as its aglycone, Cy—and correlation with the antioxidant and kinetic data. Analytical HPLC/UV-Vis Jasco (Jasco Corporation, Tokyo, Japan) equipment was used. The HPLC was equipped with a PU-2080 Plus Intelligent Pump, LG-2080-04 gradient unit, LG-2080-54 4-Line Degasser, UV-2070Plus Intelligent Detector, and LC-Net II/ADC interface (all from Jasco Corporation, Tokyo, Japan). The HPLC conditions were set as follows: Nucleosil 100 C18 column (250 mm × 4 mm, 5 μm), wavelength for anthocyanin detection 525 nm, flow 1 mL/min, room temperature; the mobile phase was composed of the solvent A (0.4% TFA in distilled water) and solvent B (0.4% TFA in acetonitrile); the gradient used for analysis was 15% B (0–6 min), 15–22% B (6–20 min), 22–35% B (20–35 min), 35–15% B (35–40 min), and 15% B for 5 min [109]. The sample volume was set for 20 μL. Acquisition and handling of the data were performed using the ChromPass Chromatography Data System version 1.7.403.1 from the same manufacturer. Limit of detection (LOD) and limit of quantitation (LOQ) values were determined according to the information provided by the HPLC equipment’s instructions and were computed using the same ChromPass Chromatography Data System version 1.7.403.1 software. Thus, five chromatograms of every standard anthocyanin compounds (i.e., Cy3G and Cy) were selected and the “noise” values in the range of ~5 min (close to the corresponding peak, but without any other secondary peaks) were determined. The LOD and LOQ values were calculated as 3·”noise” and 5·”noise”, respectively. The mean LOD and LOQ values for Cy3G and Cy were 2.54 and 4.23 μg/mL, respectively, 1.31 and 2.18 μg/mL (n = 5). RT versus LogP correlation confirmed the identification of the anthocyanin compounds in berry extracts. LogP was determined using the QSAR Properties program from the HyperChem version 7.1 software package and was based on the atomic parameters, according to Ghose et al. and Viswanadhan et al. [110,111].

### 4.5. Radical Scavenging Activity of Fruit Extracts

The antioxidant activity of the berry extracts was determined using the DPPH· method according to the literature [3,65,80], with slight modifications presented below for the not-assisted and β-CD-assisted cases. The behavior (reaction) of the 0.4 mM Cy3G ethanol solution or 1:10 diluted berry extracts in the presence of 1 mM DPPH· ethanol solution was evaluated using a Camspec M501 Single-Beam spectrophotometer (Camspec Ltd., Cambridge, UK). The acquisition and handling of the UV-Vis data were performed using the UV-Vis Analyst version 4.67 software from Camspec Ltd. (Cambridge, UK). Thus, a mixture of standard solution (0.4 mM Cy3G) or 1:10 diluted berry extract and 1 mM DPPH· solution and solvent (ethanol) at a volume ratio of 1:1:4 was monitored for 15 min at 517 nm. Possible absorbance interferences for DPPH· and anthocyanins can appear. Consequently, the absorbance of blank samples (0.4 mM Cy3G or 1:10 diluted berry extract and ethanol at a volume ratio of 1:5) was subtracted. However, the absorbance of the blank samples was less than 15% from the absorbance of the sample mixtures containing DPPH· solution at the starting of monitoring. The spectrophotometric monitoring in the presence of β-CD solution was performed in a similar way. The sample (Cy3G or diluted RB extract) was mixed with aqueous 1 mM β-CD solution, 1 mM DPPH· solution, and ethanol at a volume ratio of 1:1:1:3, followed by spectrophotometric monitoring for 15 min at 517 nm.

The antioxidant activity was evaluated by determining the actual RSA (radical scavenging activity) values during the monitoring (Equation (12)).

(12) $${RSA}_{t}\left(\%\right)=\left[1-\frac{{Abs}_{t}}{{Abs}_{0}}\right]\cdot 100$$

where RSA_t_ (%) stands for the radical scavenging activity at the time t and Abs_t_ and Abs_0_ stand for the absorbance of the mixture at the time t and t = 0 s, respectively. A high RSA value at the final of monitoring period means high antioxidant activity, while the fast or slow changes in RSA values can provide information on how the antioxidant compounds in berry extracts can have a prolonged activity. These aspects were evaluated using DPPH· reaction kinetics (see below).

### 4.6. DPPH· Reaction Kinetics in the Presence of Fruit Extracts and Standard Anthocyanins

Kinetic parameters related to the reaction between DPPH· and various antioxidant compounds in berry extracts can provide information on the behavior during the radical scavenging in vivo, as well as the influence of β-CD during the reaction. The actual concentration of the DPPH· radical during the reaction can be easily monitored using spectrophotometry using the “Time scan measurement” module (the interference of anthocyanins that absorb at 517 nm was appropriately considered and the corresponding blank absorbance was subtracted; see Figure S3 in the Supplementary Materials file). In this regard, the DPPH· calibration curve in the range of 0.01 to 0.20 mM was obtained (DPPH· Concentration (in mM) versus Absorbance (at 517 nm); Equation (13), Figures S4 and S5 in the Supplementary Materials file).

(13) $${Conc}_{DPPH\cdot }\left(mM\right)=0.1044\left(\pm 0.0004\right)\cdot {Abs}_{\left(@517nm\right)}\;n=7,r=0.99996,r^{2}=0.99992,F=70829,p<10^{-5},s=0.0011$$

The variation in the DPPH· concentration during the 15 min of monitoring reveals the complex reactions of this free radical with antioxidant compounds in berry extracts, such as Cy3G. Because the reaction is “fast” up to 30 s then becomes “slower”, three time reaction ranges were considered: 0–30 s for the “fast” reaction, 30–180 s for the “slow” reaction, and 180–900 s for the “prolonged” reaction (not studied for the kinetics point of view). Five kinetic models related to the variation in the DPPH· concentration were considered, i.e., zeroth- to fourth-(pseudo-)order kinetics (Equation (14) for the general rate law). The linear plot of the integrated rate law (integrated rate law for nth-order reaction, Equation (15)) allows us to determine the reaction rate constant, k_n_, and, further, the half-time, t_1/2_ (Equation (16)), of the DPPH· for the selected time range.

(14) $$-\frac{d\left[C\right]}{dt}=k\cdot {\left[C\right]}^{n}$$

(15) $$\frac{1}{{\left[C\right]}^{-g}}-\frac{1}{{\left[C\right]}_{0}^{-g}}=-g\cdot k\cdot t,\;for\;n\neq 1,g=1-n,\langle k\rangle =\frac{1}{{mM}^{-g}\cdot s}\;ln\left(\frac{\left[C\right]}{{\left[C\right]}_{0}}\right)=-k\cdot t,\;for\;n=1$$

(16) $$t_{1/2}=\frac{1-2^{-g}}{g}\cdot \frac{1}{k\cdot {\left[C\right]}_{0}^{-g}},\;for\;n\neq 1,g=1-n,t_{1/2}=\frac{ln\left(2\right)}{k},for\;n=1,\langle t_{1/2}\rangle =s$$

### 4.7. Statistical Analysis

Extractions and spectrophotometric determinations were made at least as duplicates. Results are presented as mean ± standard deviation, SD (unless otherwise stated), which were determined using the Basic Statistics module in the Statistica version 7.1 software package (StatSoft Inc., Tulsa, OK, USA) and Microsoft^®^ Excel^®^ 2016 in Microsoft Office Professional Plus 2016 (Microsoft Corporation, Redmond, WA, USA). Linear regression analysis (calibration curves and integrated rate law linear regression) was performed using the Multiple Linear Regression module, while the non-linear regression was performed using the Generalized Nonlinear Models module in Statistica version 7.1 package. The quality of the regression analysis was evaluated using the statistical parameters for the equation (Pearson correlation coefficient, r, determination coefficient, r^2^, adjusted determination coefficient, r^2^_adj_, Fisher and p values, and standard error of estimate, s) and for the raw correlation coefficients (standard error of the coefficient). On the other hand, the representation of the results for multiple determinations was performed using the Means with Error Plots module in the Statistica version 7.1 package, using the Whiskers graph type, Time as the grouping variable, and values as mean ± 0.95·standard error (vertical bars).

## 5. Conclusions

For the first time, the application of β-CD for enhancing the quality of the air-dehydrated berries was studied and, further, the modulation of the interactions between free radicals and antioxidants in berry extracts by β-CD was proved. In both cases, antioxidant activity of SB and RB extracts were significantly higher in the presence of β-CD. However, the best results for free radical reaction kinetics were obtained for higher-(pseudo-)order kinetic models (third- and fourth-(pseudo-)order, related to the free radical concentration). By comparison, pure anthocyanins provide good results for lower-(pseudo-)order kinetics, which is in line with other published kinetic studies. This difference can be due to the complexity of parallel and consecutive reactions involving antioxidants in extracts and free radicals, especially in the presence of β-CD that forms molecular inclusion compounds with these bioactive compounds. Finally, the protection capacity of β-CD against the degradation of antioxidants during the dehydration process of berries was proved. The technology can be extended to other fruits and scale up for obtaining high-quality fruit-based products.

## Figures and Tables

**Table 1:** Cyanidin 3-*O*-glucoside (Cy3G), cyanidin (Cy), and total anthocyanin and anthocyanidin content (as Cy3G) in fresh raspberries (values are expressed as mean ± SD, n = 5).

Anthocyanins and Anthocyanidins	Content (mg/100 g fw)
Cyanidin 3-*O*-glucoside	14.62 ± 2.64
Cyanidin	0.64 ± 0.07
Total anthocyanins and anthocyanidins ^1^	73.18 ± 12.39

^1^ Total anthocyanin and anthocyanidin content was determined by HPLC (all antioxidant compounds separated and identified at 525 nm were counted and the content was expressed as mg cyanidin 3-*O*-glucoside/100 g fw).

**Table 2:** Cyanidin 3-*O*-glucoside (Cy3G), pelargonidin 3-*O*-glucoside (Plg3G), cyanidin (Cy), and total anthocyanin and anthocyanidin content (as mg Cy3G/100 g fw) in fresh strawberries (values are expressed as mean ± SD, n = 2).

Anthocyanins and Anthocyanidins	Content in Fresh Fruits (mg/100 g fw)	Content in β-CD-Assisted Dehydrated Fruits (mg/100 g) ^1^
Cyanidin 3-*O*-glucoside	0.32 ± 0.08	0.15
Pelargonidin 3-*O*-glucoside ^2^	2.46 ± 0.38	1.64
Cyanidin	0.017 ± 0.003	0.033
Total anthocyanins and anthocyanidins ^3^	4.28 ± 0.56	4.30

^1^ Single determination. ^2^ Pelargonidin 3-*O*-glucoside was identified according to [18,19,20] and was quantified as mg Cy3G/100 g. ^3^ Total anthocyanin and anthocyanidin content was determined by HPLC (all antioxidant compounds separated and identified at 525 nm were counted, and the content is expressed as mg Cy3G/100 g).

**Table 3:** Representative Radical Scavenging Activity (RSA) values for the fresh strawberry extracts, 1:10 diluted fresh strawberry extracts (SB and SB_d1:10), and undiluted and 1:10 diluted extracts obtained from the dehydrated strawberries without (SBD and SBD_d1:10) and with β-cyclodextrin (SBD_bCD and SBD_bCD_d1:10) as an additive. For undiluted samples, RSA values with different superscript capital letters are significantly different according to Tukey’s HSD (honestly significant difference) test (p < 0.01; on the same column). For 1:10 diluted samples, RSA values with different superscript lowercase letters are significantly different according to Tukey’s HSD test (p < 0.01; on the same column).

Sample Code	RSA (%) (@ ½ min)	RSA (%) (@ 3 min)	RSA (%) (@ 15 min)
SB	44.84 ± 9.59 ^A^	67.80 ± 8.10 ^A^	81.17 ± 2.20 ^A^
SB_d1:10	4.37 ± 1.31 ^a^	8.60 ± 1.68 ^a^	13.40 ± 1.54 ^a^
SBD *	81.47 ^A^	82.46 ^A^	82.53 ^A^
SBD_d1:10 *	13.04 ^b^	22.96 ^b^	35.14 ^b^
SBD_bCD *	83.98 ^A^	84.32 ^A^	84.45 ^A^
SBD_bCD_d1:10 *	19.19 ^c^	31.97 ^c^	47.32 ^c^

* Single determination.

**Table 4:** Representative Radical Scavenging Activity (RSA) values for the fresh raspberry extracts not assisted (RB) or β-cyclodextrin assisted (RB_bCD) during the spectrophotometric monitoring (values are presented as mean ± standard deviation, SD, for duplicate samples). In the same column, RSA values with different superscript lowercase letters are significantly different according to Tukey’s HSD test (p < 0.1 for the RSA values at ½ and 3 min, respectively, p < 0.05 for the RSA values at 15 min).

Sample Code	RSA (%) (@ ½ min)	RSA (%) (@ 3 min)	RSA (%) (@ 15 min)
RB	18.22 ± 3.67 ^a^	46.96 ± 2.83 ^a^	72.93 ± 0.99 ^c^
RB_bCD	26.15 ± 0.52 ^b^	55.67 ± 1.31 ^b^	77.41 ± 0.58 ^d^

**Table 5:** Representative Radical Scavenging Activity (RSA) values for the standard 0.4 mM cyanidin 3-*O*-glucoside not assisted (Cy3G0.4mM) or β-cyclodextrin assisted (Cy3G&bCD) during the spectrophotometric monitoring (values are presented as mean ± standard deviation, SD, for duplicate samples). In the same column, RSA values with different superscript lowercase letters are significantly different according to Tukey’s HSD test (p < 0.1 for the RSA values at 3 min, respectively, p < 0.15 for the RSA values at 15 min).

Sample Code	RSA (%) (@ ½ min)	RSA (%) (@ 3 min)	RSA (%) (@ 15 min)
Cy3G0.4mM	20.92 ± 9.42 ^a^	37.76 ± 12.41 ^a^	66.37 ± 13.37 ^c^
Cy3G&bCD	29.13 ± 0.49 ^a^	65.35 ± 0.21 ^b^	89.63 ± 0.90 ^d^

## Data Availability

Data is contained within the article or Supplementary Materials file.

## Supplementary Materials

The following supporting information can be downloaded at https://www.mdpi.com/article/10.3390/plants15010152/s1. Figure S1: Concentration (mg/mL) versus HPLC peak area (mV·min) calibration curve for the cyanidin 3-*O*-glucoside standard solutions in the range of 0.05–0.50 mg/mL; Figure S2: Concentration (mg/mL) versus HPLC peak area (mV·min) calibration curve for the cyanidin standard solutions in the range of 0.025–0.50 mg/mL; Figure S3: Comparison between the UV-Vis spectra (range of 400–700 nm) of the cyanidin 3-*O*-glucoside (Cy3G) standard solution (e.g., 0.06 mM—blue) and the standard DPPH· solutions (e.g., from bottom to top: 0.01, 0.02 and 0.16 mM, which is the working concentration at the start of the measurements—green). The Cy3G standard solution has a maximum absorbance in the range of the high diluted DPPH· solutions of 0.01 and 0.02 mM. However, the Cy3G working concentration at the start of the measurement is four times lower (0.015 mM). The spectra for the blank sample (ethanol) is in red; Figure S4: UV-Vis spectra (region 400–700 nm) of the DPPH· standard solutions (from bottom to top, concentrations from 0.01, 0.02, 0.04, 0.08, 0.12, 0.16 and 0.20 mM). The spectra for the blank sample (ethanol) is in red; Figure S5: Concentration (mM) versus Absorbance (@517 nm) calibration curve for the DPPH· standard solutions in the range of 0.01–0.20 mM; Figure S6: UV-Vis monitoring (at 517 nm) of the 0.4 mM cyanidin 3-*O*-glucoside and 1 mM DPPH· solutions in ethanol, at a volume ratio of 1:1:4 (duplicate samples; blank ethanol is in red); Figure S7: UV-Vis monitoring (at 517 nm) of the 0.4 mM cyanidin 3-*O*-glucoside, 1 mM DPPH· and 1 mM β-cyclodextrin (in water) solutions, mixed with ethanol at a volume ratio of 1:1:1:3 (duplicate samples; blank ethanol is in red); Figure S8: Superimposed Absorbance versus Time plots from the UV-Vis monitoring (at 517 nm) of the 0.4 mM cyanidin 3-*O*-glucoside and 1 mM DPPH· solutions in ethanol at 1:1:4 volume ratio (duplicate samples; blue colors) and for the 0.4 mM cyanidin 3-*O*-glucoside, 1 mM DPPH· and 1 mM β-cyclodextrin (in water) solutions, mixed with ethanol at a volume ratio of 1:1:1:3 (duplicate samples; green colors) (blank ethanol is in red, blank cyanidin 3-*O*-glucoside solution is in purple and blank DPPH· solution is in black); Figure S9: Antioxidant activity of the fresh strawberry extracts, expressed as the variation in the Radical Scavenging Activity (RSA, %) in Time (s) for 15 min, as well as the logarithmic correlations (duplicate samples); Figure S10: Antioxidant activity of the fresh strawberry extracts (1:10 dilutions), expressed as the variation in the Radical Scavenging Activity (RSA, %) in Time (s) for 15 min, as well as the logarithmic correlations (duplicate samples); Figure S11: Antioxidant activity of the extracts obtained from dehydrated strawberries, without (blue) and with β-cyclodextrin (red) as additive (1:10 dilutions). Antioxidant activity is expressed as the variation in the Radical Scavenging Activity (RSA, %) in Time (s) for 15 min. Logarithmic correlations are also provided; Figure S12: Antioxidant activity of the extracts obtained from fresh (blue) and dehydrated strawberries, without (red) and with β-cyclodextrin (green) as additive (1:10 dilutions). Antioxidant activity is expressed as the variation in the Radical Scavenging Activity (RSA, %) in Time (s) for 15 min. Logarithmic correlations are also provided; Figure S13: Antioxidant activity of the extracts obtained from fresh raspberries, β-cyclodextrin assisted (green diamond) or not assisted (brown square) during the spectrophotometric monitoring (duplicate samples). Antioxidant activity is expressed as the variation in the Radical Scavenging Activity (RSA, %) in Time (s) for 15 min. For multiple determinations, Whisker type plots as mean ± 0.95·standard error were used. Logarithmic correlations are also provided; Figure S14: Antioxidant activity of the standard 0.4 mM cyanidin 3-*O*-glucoside, β-cyclodextrin assisted (green diamond) or not assisted (brown square) during the spectrophotometric monitoring (duplicate samples). Antioxidant activity is expressed as the variation in the Radical Scavenging Activity (RSA, %) in Time (s) for 15 min. For multiple determinations, Whisker type plots as mean ± 0.95·standard error were used. Logarithmic correlations are also provided; Figure S15: Integrated rate law representation for the zeroth–fourth-order models for the “fast” DPPH· reaction with antioxidant compounds from the extracts obtained from the fresh and dehydrated strawberries (without or with β-cyclodextrin as additive; 1:10 diluted extracts). For duplicate samples, Whisker type plots as mean ± 0.95·standard error were used; Figure S16: Integrated rate law representation for the zeroth–fourth-order models for the “slow” DPPH· reaction with antioxidant compounds from the extracts obtained from the fresh and dehydrated strawberries (without or with β-cyclodextrin as additive; 1:10 diluted extracts). For duplicate samples, Whisker type plots as mean ± 0.95·standard error were used; Figure S17: Integrated rate law representation for the zeroth–fourth-order models for the “fast” DPPH· reaction with antioxidant compounds from the extracts obtained from the fresh raspberries (without or with β-cyclodextrin as additive during the spectrophotometric monitoring). For duplicate samples, Whisker type plots as mean ± 0.95·standard error were used; Figure S18: Integrated rate law representation for the zeroth–fourth-order models for the “slow” DPPH· reaction with antioxidant compounds from the extracts obtained from the fresh raspberries (without or with β-cyclodextrin as additive during the spectrophotometric monitoring). For duplicate samples, Whisker type plots as mean ± 0.95·standard error were used; Figure S19: Integrated rate law representation for the zeroth–fourth-order models for the “fast” DPPH· reaction with the standard 0.4 mM cyanidin 3-*O*-glucoside (without or with β-cyclodextrin as additive during the spectrophotometric monitoring). For duplicate samples, Whisker type plots as mean ± 0.95·standard error were used; Figure S20: Integrated rate law representation for the zeroth–fourth-order models for the “slow” DPPH· reaction with the standard 0.4 mM cyanidin 3-*O*-glucoside (without or with β-cyclodextrin as additive during the spectrophotometric monitoring). For duplicate samples, Whisker type plots as mean ± 0.95·standard error were used; Figure S21: Isosurface image for the most stable conformation of the cyanidin 3-*O*-glucoside (Cy3G). The total charge density was in the range of −0.3 to 0.3 (mapped function range) and is shown in the upper-left corner as a range legend; Figure S22: Isosurface image for the most stable conformation of the cyanidin (Cy). The total charge density was in the range of −0.3 to 0.3 (mapped function range) and is shown in the upper-left corner as a range legend; Figure S23: Isosurface image for the most stable conformation of the pelargonidin 3-*O*-glucoside (Plg3G). The total charge density was in the range of −0.3 to 0.3 (mapped function range) and is shown in the upper-left corner as a range legend; Figure S24: Optimized β-cyclodextrin/DPPH· complex, obtained with MM+ Molecular Mechanics Force Field module and Polak-Ribiere (Conjugate gradient) algorithm from the HyperChem 7.52 software package. Both β-CD and DPPH· were built and optimized separately as was presented above. The interaction starts with the DPPH· structure (both the Ph_2_ and picryl sides) oriented to the secondary face of the β-CD, at a distance of ~8 Å between the gravity centers. DPPH· molecule is highlighted. The picryl moiety is only partial encapsulated into the β-CD cavity (at approximately 50%); Figure S25: Optimized β-cyclodextrin/cyanidin 3-*O*-glucoside complex, obtained with MM+ Molecular Mechanics Force Field module and Polak-Ribiere (Conjugate gradient) algorithm from the HyperChem 7.52 software package. Both β-CD and Cy3G were built and optimized separately as was presented above. The interaction starts with the Cy3G structure (benzopyrane moiety) oriented to the secondary face of the β-CD, at a distance of ~8 Å between the gravity centers. Cy3G molecule is highlighted. Benzo moiety is completely encapsulated. The 2-phenyl moiety (not encapsulated) has the 3- and 4-OH groups oriented to the solution, as well as the glucose moiety. This favors the reaction with the DPPH· radical from the solution; Table S1: Kinetic and statistic parameters corresponding to the zeroth–fourth-order models for the “fast” DPPH· reaction with antioxidant compounds from the 1:10 diluted extracts obtained from the fresh and dehydrated strawberries (without or with β-cyclodextrin as additive). For 1:10 diluted samples, k values with different superscript capital letters are significantly different, according to Tukey’s HSD test (p < 0.01; on the same raw). For 1:10 diluted samples, t_1/2_ values with different superscript lowercase letters are significantly different, according to Tukey’s HSD test (p < 0.05; on the same raw); Table S2: Kinetic and statistic parameters corresponding to the zeroth–fourth-order models for the “slow” DPPH· reaction with antioxidant compounds from the 1:10 diluted extracts obtained from the fresh and dehydrated strawberries (without or with β-cyclodextrin as additive). For 1:10 diluted samples, k values with different superscript capital letters are significantly different, according to Tukey’s HSD test (p < 0.001; on the same raw). For 1:10 diluted samples, t_1/2_ values with different superscript lowercase letters are significantly different, according to Tukey’s HSD test (p < 0.01; on the same raw); Table S3: Kinetic and statistic parameters corresponding to the zeroth–fourth-order models for the “fast” DPPH· reaction with antioxidant compounds from the extracts obtained from the fresh raspberries (without or with β-cyclodextrin as additive during the spectrophotometric monitoring). On the same row, k values with different superscript capital letters are significantly different, according to Tukey’s HSD test (p < 0.10). On the same row, t_1/2_ values with different superscript lowercase letters are significantly different, according to Tukey’s HSD test (p < 0.15); Table S4: Kinetic and statistic parameters corresponding to the zeroth–fourth-order models for the “slow” DPPH· reaction with antioxidant compounds from the extracts obtained from the fresh raspberries (without or with β-cyclodextrin as additive during the spectrophotometric monitoring). On the same row, k values with different superscript capital letters are significantly different, according to Tukey’s HSD test (p < 0.05). On the same row, t_1/2_ values with different superscript lowercase letters are significantly different, according to Tukey’s HSD test (p < 0.05); Table S5: Kinetic and statistic parameters corresponding to the zeroth–fourth-order models for the “fast” DPPH· reaction with standard 0.4 mM cyanidin 3-*O*-glucoside (without or with β-cyclodextrin as additive during the spectrophotometric monitoring). On the same row, k values with different superscript capital letters are significantly different, according to Tukey’s HSD test (p < 0.05). On the same row, t_1/2_ values with different superscript lowercase letters are significantly different, according to Tukey’s HSD test (p < 0.05); Table S6: Kinetic and statistic parameters corresponding to the zeroth–fourth-order models for the “slow” DPPH· reaction with standard 0.4 mM cyanidin 3-*O*-glucoside (without or with β-cyclodextrin as additive during the spectrophotometric monitoring). On the same row, k values with different superscript capital letters are significantly different, according to Tukey’s HSD test (p < 0.05). On the same row, t_1/2_ values with different superscript lowercase letters are significantly different, according to Tukey’s HSD test (p < 0.15).

## Abbreviations

The following abbreviations are used in this manuscript:

α-CD	α-Cyclodextrin
Abs	Absorbance (in UV-Vis spectrophotometry)
ABTS^+^·	2,2′-Azino-bis(3-ethylbenzothiazoline-6-sulfonic acid) radical cation
AP	Antioxidant power
ASA	Acetylsalicylic acid
β-CD	β-Cyclodextrin
CD	Cyclodextrin
Cy3G	Cyanidin 3-*O*-glucopyranoside
DPPH·	2,2-Diphenyl-1-picrylhydrazyl radical
dw	dry weight
EC_50_	Half maximal effective concentration
F	Fisher-value (Fisher-test)
FRAP	Ferric reducing antioxidant power
γ-CD	γ-Cyclodextrin
GRAS	Generally recognized as safe
GSH	Glutathione
HSD	Honestly significant difference (Tukey’s HSD test)
k_n_	Reaction rate constant for the nth order kinetic model
HPLC	High pressure liquid chromatography
LOD	Limit of detection
LogP	Logarithm of the 1-octanol/water partition coefficient
LOQ	Limit of quantitation
M	Molar concentration, mol/L
mM	milimolar concentration, mmol/L
NaDES	Natural deep eutectic solvent
ORAC	Oxygen radical absorbance capacity
p-value	probability (null-hypothesis significance test)
PEF	Pulsed electric field
Plg3G	Pelargonidin 3-*O*-glucopyranoside
QSAR	Quantitative structure-activity relationship
r/r^2^	Correlation/determination coefficient
RB	Raspberry (*Rubus idaeus* L.)
RP-HPLC	Reversed phase—high pressure liquid chromatography
RSA	Radical scavenging activity
RT	Retention time
s	standard error of estimate
SB	Strawberry (*Fragaria* × *ananassa* Duch.)
SD	Standard deviation
t_1/2(n)_	Half-time for the nth order kinetic model
TAC	Total anthocyanin content
TE	Trolox equivalent
TEAC	Trolox equivalent antioxidant capacity
TFA	Trifluoroacetic acid
TPC	Total phenolic content
UV-Vis	Ultraviolet-visible spectrophotometry

## References

[1] Neha K., Haider M.R., Pathak A., Yar M.S. (2019). Medicinal prospects of antioxidants: A review. Eur. J. Med. Chem..

[2] Mohammed D.M., Maan S.A., Baker D.H.A., Abozed S.S. (2024). In vitro assessments of antioxidant, antimicrobial, cytotoxicity and anti-inflammatory characteristics of flavonoid fractions from flavedo and albedo orange peel as novel food additives. Food Biosci..

[3] Hussain A., Batool A., Yaqub S., Iqbal A., Kauser S., Arif M.R., Ali S., Gorsi F.I., Nisar R., Hussain A. (2024). Effects of spray drying and ultrasonic assisted extraction on the phytochemicals, antioxidant and antimicrobial activities of strawberry fruit. Food Chem. Adv..

[4] Silva F.K.d.S., Costa-Orlandi C.B., Fernandes M.A., Brasil G.S.A.P., Mussagy C.U., Scontri M., Sasaki J.C.d.S., Abreu A.P.d.S., Guerra N.B., Floriano J.F. (2023). Biocompatible anti-aging face mask prepared with curcumin and natural rubber with antioxidant properties. Int. J. Biol. Macromol..

[5] Oyenihi A.B., Smith C. (2019). Are polyphenol antioxidants at the root of medicinal plant anti-cancer success?. J. Ethnopharmacol..

[6] Wang S., Meckling K.A., Marcone M.F., Kakuda Y., Tsao R. (2011). Can phytochemical antioxidant rich foods act as anti-cancer agents?. Food Res. Int..

[7] Mao X.-Y., Jin M.-Z., Chen J.-F., Zhou H.-H., Jin W.-L. (2018). Live or let die: Neuroprotective and anti-cancer effects of nutraceutical antioxidants. Pharmacol. Ther..

[8] Masaki H. (2010). Role of antioxidants in the skin: Anti-aging effects. J. Dermatol. Sci..

[9] Ghorbani A. (2017). Mechanisms of antidiabetic effects of flavonoid rutin. Biomed. Pharmacother..

[10] Tolmie M., Bester M.J., Serem J.C., Nell M., Apostolides Z. (2023). The potential antidiabetic properties of green and purple tea [*Camellia sinensis* (L.) O Kuntze], purple tea ellagitannins, and urolithins. J. Ethnopharmacol..

[11] Ali A., Cottrell J.J., Dunshea F.R. (2022). Identification and characterization of anthocyanins and non-anthocyanin phenolics from Australian native fruits and their antioxidant, antidiabetic, and anti-Alzheimer potential. Food Res. Int..

[12] Zhu M., Bao J., Liang P., Zhang C., Li S., Hu F. (2025). Extraction of flavonoids and phenolic acids from poplar-type propolis: Optimization of maceration process, evaluation of antioxidant and antimicrobial activities. LWT—Food Sci. Technol..

[13] Zhou P., Pan Y., Yang W., Yang B., Ou S., Liu P., Zheng J. (2023). Hepatoprotective effect of cyanidin-3-*O*-glucoside–lauric acid ester against H_2_O_2_-induced oxidative damage in LO_2_ cells. J. Funct. Foods.

[14] Bwambale W., Oka V.O., Etukudo E.M., Owu D.U., Nkanu E.E., Okon I.A., Emmanuel S.D., Shehu U.U., Wilber N., Kambere A. (2025). Nephroprotective activity of naringenin in gentamicin-induced nephrotoxicity in male Wistar rats: In-vivo and in-silico evaluation. Pharmacol. Res.—Nat. Prod..

[15] Martínez-Busi M., Arredondo F., González D., Echeverry C., Vega-Teijido M.A., Carvalho D., Rodríguez-Haralambides A., Rivera F., Dajas F., Abin-Carriquiry J.A. (2019). Purification, structural elucidation, antioxidant capacity and neuroprotective potential of the main polyphenolic compounds contained in *Achyrocline satureioides* (Lam) D.C. (Compositae). Bioorganic Med. Chem..

[16] Kruger M.J., Davies N., Myburgh K.H., Lecour S. (2014). Proanthocyanidins, anthocyanins and cardiovascular diseases. Food Res. Int..

[17] Mladěnka P., Zatloukalová L., Filipský T., Hrdina R. (2010). Cardiovascular effects of flavonoids are not caused only by direct antioxidant activity. Free Radic. Biol. Med..

[18] Canuto G.A.B., Oliveira D.R., da-Conceição L.S.M., Farah J.P.S., Tavares M.F.M. (2016). Development and validation of a liquid chromatography method for anthocyanins in strawberry (*Fragaria* spp.) and complementary studies on stability, kinetics and antioxidant power. Food Chem..

[19] Chaves V.C., Calvete E., Reginatto F.H. (2017). Quality properties and antioxidant activity of seven strawberry (*Fragaria* × *ananassa* duch) cultivars. Sci. Hortic..

[20] Dzhanfezova T., Barba-Espín G., Müller R., Joernsgaard B., Hegelund J.N., Madsen B., Larsen D.H., Vega M.M., Toldam-Andersen T.B. (2020). Anthocyanin profile, antioxidant activity and total phenolic content of a strawberry (*Fragaria* × *ananassa* Duch) genetic resource collection. Food Biosci..

[21] Nowicka A., Kucharska A.Z., Sokół-Łętowska A., Fecka I. (2019). Comparison of polyphenol content and antioxidant capacity of strawberry fruit from 90 cultivars of *Fragaria* × *ananassa* Duch. Food Chem..

[22] Hădărugă D.I., Hădărugă N.G., Jafari S.M., Rashidinejad A., Simal-Gandara J. (2023). Flavonols. Chemistry, Functionality, and Applications. Handbook of Food Bioactive Ingredients: Properties and Applications.

[23] Hădărugă N.G., Hădărugă D.I., Jafari S.M., Rashidinejad A., Simal-Gandara J. (2023). Hydroxycinnamic acids. Handbook of Food Bioactive Ingredients: Properties and Applications.

[24] Bobinaitė R., Viškelis P., Venskutonis P.R., Simmonds M.S.J., Preedy V.R. (2016). Nutritional Composition of Fruit Cultivars. Nutritional Composition of Fruit Cultivars.

[25] de-Souza V.R., Pereira P.A.P., da-Silva T.L.T., Lima L.C.d.O., Pio R., Queiroz F. (2014). Determination of the bioactive compounds, antioxidant activity and chemical composition of Brazilian blackberry, red raspberry, strawberry, blueberry and sweet cherry fruits. Food Chem..

[26] Drobek M., Cybulska J., Zdunek A., Sas-Paszt L., Frąc M. (2024). Effect of microbial biostimulants on the antioxidant profile, antioxidant capacity and activity of enzymes influencing the quality level of raspberries (*Rubus idaeus* L.). Food Chem..

[27] Sinha N.K., Sinha N.K., Sidhu J.S., Barta J., Wu J.S.B., Cano M.P. (2012). Strawberries and Raspberries. Handbook of Fruits and Fruit Processing.

[28] Wang Y., Ye Y., Wang L., Yin W., Liang J. (2021). Antioxidant activity and subcritical water extraction of anthocyanin from raspberry process optimization by response surface methodology. Food Biosci..

[29] de-Souza V.B., Fujita A., Thomazini M., da-Silva E.R., Francisco Lucon J., Genovese M.I., Favaro-Trindade C.S. (2014). Functional properties and stability of spray-dried pigments from Bordo grape (*Vitis labrusca*) winemaking pomace. Food Chem..

[30] Krgović N., Jovanović M.S., Nedeljković S.K., Šavikin K., Lješković N.J., Ilić M., Živković J., Menković N. (2025). Natural deep eutectic solvents extraction of anthocyanins—Effective method for valorisation of black raspberry (*Rubus occidentalis* L.) pomace. Ind. Crops Prod..

[31] Avalos-Llano K.R., Martín-Belloso O., Soliva-Fortuny R. (2018). Effect of pulsed light treatments on quality and antioxidant properties of fresh-cut strawberries. Food Chem..

[32] Lucan-(Banciu) C.A., Cugerean M.I., Gălan I.M., Ciobanu-(Şibu) A., Pop-(Mateuţ) A., Oprinescu C.I., Drăghici L.R., Hădărugă D.I., Hădărugă N.G. (2024). State-of-the-art on the Dehydration and Drying of *Cucurbita* Species. Bull. Univ. Agric. Sci. Vet. Med. Cluj-Napoca—Food Sci. Technol..

[33] Asioli D., Rocha C., Wongprawmas R., Popa M., Gogus F., Almli V.L. (2019). Microwave-dried or air-dried? Consumers’ stated preferences and attitudes for organic dried strawberries. A multi-country investigation in Europe. Food Res. Int..

[34] Amami E., Khezami W., Mezrigui S., Badwaik L.S., Bejar A.K., Perez C.T., Kechaou N. (2017). Effect of ultrasound-assisted osmotic dehydration pretreatment on the convective drying of strawberry. Ultrason. Sonochemistry.

[35] de-Bruijn J., Bórquez R. (2014). Quality retention in strawberries dried by emerging dehydration methods. Food Res. Int..

[36] Contreras C., Martín-Esparza M.E., Chiralt A., Martínez-Navarrete N. (2008). Influence of microwave application on convective drying: Effects on drying kinetics, and optical and mechanical properties of apple and strawberry. J. Food Eng..

[37] Moreno J., Simpson R., Pizarro N., Parada K., Pinilla N., Reyes J.E., Almonacid S. (2012). Effect of ohmic heating and vacuum impregnation on the quality and microbial stability of osmotically dehydrated strawberries (cv. Camarosa). J. Food Eng..

[38] Adak N., Heybeli N., Ertekin C. (2017). Infrared drying of strawberry. Food Chem..

[39] Avcı A.S., Çetinbaş A., Karakaya H. (2025). Analysis of strawberry drying behavior under a solar chimney collector using a full factorial method. Case Stud. Therm. Eng..

[40] Doymaz İ. (2008). Convective drying kinetics of strawberry. Chem. Eng. Process..

[41] El-Beltagy A., Gamea G.R., Essa A.H.A. (2007). Solar drying characteristics of strawberry. J. Food Eng..

[42] Jiang D.-L., Wang Q.-H., Huang C., Sutar P.P., Lin Y.-W., Okaiyeto S.A., Lin Z.-F., Wu Y.-T., Ma W.-M., Xiao H.-W. (2024). Effect of various different pretreatment methods on infrared combined hot air impingement drying behavior and physicochemical properties of strawberry slices. Food Chem. X.

[43] Zambon A., Zulli R., Boldrin F., Spilimbergo S. (2022). Microbial inactivation and drying of strawberry slices by supercritical CO_2_. J. Supercrit. Fluids.

[44] Kowalski S.J., Pawłowski A., Szadzińska J., Łechtańska J., Stasiak M. (2016). High power airborne ultrasound assist in combined drying of raspberries. Innov. Food Sci. Emerg. Technol..

[45] Li S., Luo S., Wang H., Liu H., Liu J., Zhang X., Tian B. (2025). Chitosan/polyvinyl alcohol film loading β-acids/β-cyclodextrin inclusion complex: A shelf-life extension strategy for strawberry. Int. J. Biol. Macromol..

[46] Lu H., Li S., Gao Q. (2025). Development of an antibacterial hydrogel by κ-carrageenan with carvacrol/hydroxypropyl-β-cyclodextrin composite and its application for strawberry preservation. Food Control.

[47] Peinado I., Rosa E., Heredia A., Escriche I., Andrés A. (2016). Influence of storage on the volatile profile, mechanical, optical properties and antioxidant activity of strawberry spreads made with isomaltulose. Food Biosci..

[48] Zhang Y., Li F., Yan H., Zhang Y., Feng S., Deng L., Zhao L., Gao S., Fu Y., Ye F. (2025). Nerolidol/hydroxypropyl-*beta*-cyclodextrin inclusion complex nanofibers: Active food packaging for effective strawberry preservation. Food Chem. X.

[49] Fan Y., Xu Y., Wang D., Zhang L., Sun J., Sun L., Zhang B. (2009). Effect of alginate coating combined with yeast antagonist on strawberry (*Fragaria* × *ananassa*) preservation quality. Postharvest Biol. Technol..

[50] Huang L.-L., Zhang M., Yan W.-Q., Mujumdar A.S., Sun D.-F. (2009). Effect of coating on post-drying of freeze-dried strawberry pieces. J. Food Eng..

[51] Wang H., Yuan D., Meng Q., Zhang Y., Kou X., Ke Q. (2024). Pickering nanoemulsion loaded with eugenol contributed to the improvement of konjac glucomannan film performance. Int. J. Biol. Macromol..

[52] Cerezo A.B., Cuevas E., Winterhalter P., Garcia-Parrilla M.C., Troncoso A.M. (2010). Isolation, identification, and antioxidant activity of anthocyanin compounds in *Camarosa* strawberry. Food Chem..

[53] Agudelo-Laverde L.M., Schebor C., Buera M.d.P. (2013). Water content effect on the chromatic attributes of dehydrated strawberries during storage, as evaluated by image analysis. LWT—Food Sci. Technol..

[54] Bórquez R.M., Canales E.R., Redon J.P. (2010). Osmotic dehydration of raspberries with vacuum pretreatment followed by microwave-vacuum drying. J. Food Eng..

[55] Angeli L., Morozova K., Scampicchio M. (2023). A kinetic-based stopped-flow DPPH• method. Sci. Rep..

[56] Kedare S.B., Singh R.P. (2011). Genesis and development of DPPH method of antioxidant assay. J. Food Sci. Technol..

[57] Osugi J., Sasaki M. (1964). Kinetic studies on free radical reactions. II. The photochemical reaction between DPPH and methylmethacrylate. Rev. Phys. Chem. Jpn..

[58] Sendra J.M., Sentandreu E., Navarro J.L. (2006). Reduction kinetics of the free stable radical 2,2-diphenyl-1-picrylhydrazyl (DPPH•) for determination of the antiradical activity of citrus juices. Eur. Food Res. Technol..

[59] Goupy P., Dufour C., Loonis M., Dangles O. (2003). Quantitative Kinetic Analysis of Hydrogen Transfer Reactions from Dietary Polyphenols to the DPPH Radical. J. Agric. Food Chem..

[60] Foti M.C., Daquino C., DiLabio G.A., Ingold K.U. (2011). Kinetics of the Oxidation of Quercetin by 2,2-Diphenyl-1-picrylhydrazyl (dpph•). Org. Lett..

[61] Shojaee M.S., Moeenfard M., Farhoosh R. (2022). Kinetics and stoichiometry of gallic acid and methyl gallate in scavenging DPPH radical as affected by the reaction solvent. Sci. Rep..

[62] Rothwell J.A., Day A.J., Morgan M.R.A. (2005). Experimental Determination of Octanol-Water Partition Coefficients of Quercetin and Related Flavonoids. J. Agric. Food Chem..

[63] Yanagida A., Murao H., Ohnishi-Kameyama M., Yamakawa Y., Shoji A., Tagashira M., Kanda T., Shindo H., Shibusawa Y. (2007). Retention behavior of oligomeric proanthocyanidins in hydrophilic interaction chromatography. J. Chromatogr. A.

[64] Moshari-Nasirkandi A., Alirezalu A., Hachesu M.A. (2020). Effect of lemon verbena bio-extract on phytochemical and antioxidant capacity of strawberry (*Fragaria* × *ananassa* Duch. cv. Sabrina) fruit during cold storage. Biocatal. Agric. Biotechnol..

[65] Mustafa A.M., Angeloni S., Abouelenein D., Acquaticci L., Xiao J., Sagratini G., Maggi F., Vittori S., Caprioli G. (2022). A new HPLC-MS/MS method for the simultaneous determination of 36 polyphenols in blueberry, strawberry and their commercial products and determination of antioxidant activity. Food Chem..

[66] Corrales M., Lindauer R., Butz P., Tauscher B. (2008). Effect of heat/pressure on cyanidin-3-glucoside ethanol model solutions. J. Phys. Conf. Ser..

[67] Zhang Y., Sun J., Hu X., Liao X. (2010). Spectral Alteration and Degradation of Cyanidin-3-glucoside Exposed to Pulsed Electric Field. J. Agric. Food Chem..

[68] Ichiyanagi T., Oikawa K., Tateyama C., Konishi T. (2001). Acid Mediated Hydrolysis of Blueberry Anthocyanins. Chem. Pharm. Bull..

[69] Sadilova E., Carle R., Stintzing F.C. (2007). Thermal degradation of anthocyanins and its impact on color and in vitro antioxidant capacity. Mol. Nutr. Food Res..

[70] Hădărugă D.I., Hădărugă N.G., Jafari S.M., Rashidinejad A., Simal-Gandara J. (2023). Flavanones in Plants and Humans. Chemistry, Functionality, and Applications. Handbook of Food Bioactive Ingredients: Properties and Applications.

[71] Hădărugă N.G., Hădărugă D.I., Jafari S.M., Rashidinejad A., Simal-Gandara J. (2023). Stilbenes and Its Derivatives and Glycosides. Handbook of Food Bioactive Ingredients: Properties and Applications.

[72] Hădărugă D.I., Hădărugă N.G., Bandur G.N., Isengard H.-D. (2012). Water content of flavonoid/cyclodextrin nanoparticles: Relationship with the structural descriptors of biologically active compounds. Food Chem..

[73] Hădărugă N.G., Bandur G.N., David I., Hădărugă D.I. (2019). A review on thermal analyses of cyclodextrins and cyclodextrin complexes. Environ. Chem. Lett..

[74] Mandave P.C., Pawar P.K., Ranjekar P.K., Mantri N., Kuvalekar A.A. (2014). Comprehensive evaluation of in vitro antioxidant activity, total phenols and chemical profiles of two commercially important strawberry varieties. Sci. Hortic..

[75] Rahman M.H., Hasan M.N., Khan M.Z.H. (2021). Study on different nano fertilizers influencing the growth, proximate composition and antioxidant properties of strawberry fruits. J. Agric. Food Res..

[76] Wang J., Wang J., Ye J., Vanga S.K., Raghavan V. (2019). Influence of high-intensity ultrasound on bioactive compounds of strawberry juice: Profiles of ascorbic acid, phenolics, antioxidant activity and microstructure. Food Control.

[77] Huang X., Wu Y., Zhang S., Yang H., Wu W., Lyu L., Li W. (2023). Changes in antioxidant substances and antioxidant enzyme activities in raspberry fruits at different developmental stages. Sci. Hortic..

[78] Zhang L., Li J., Hogan S., Chung H., Welbaum G.E., Zhou K. (2010). Inhibitory effect of raspberries on starch digestive enzyme and their antioxidant properties and phenolic composition. Food Chem..

[79] Falah M.A.F., Machfoedz M.M., Rahmatika A.M., Putri R.M. (2025). Quality characterization of freeze-dried tropical strawberries pretreated through osmotic dehydration. J. Agric. Food Res..

[80] Santos N.C., Almeida R.L.J., Monteiro S.S., Silva E.T.d.V., Silva V.M.d.A., André A.M.M.C.N., Ribeiro V.H.d.A., de-Brito A.C.O. (2022). Influence of ethanol and ultrasound on drying, bioactive compounds, and antioxidant activity of strawberries (*Fragaria* × *ananassa*). J. Indian Chem. Soc..

[81] Chat O.A., Najar M.H., Dar A.A. (2013). Evaluation of reduction kinetics of 2,2-diphenyl-1-picrylhydrazylradical by flavonoid glycoside Rutin in mixed solvent based micellar media. Colloids Surf. A Physicochem. Eng. Asp..

[82] Foti M.C., Slavova-Kazakov A., Rocco C., Kancheva V.D. (2016). Kinetics of curcumin oxidation by 2,2-diphenyl-1-picrylhydrazyl (DPPH˙): An interesting case of separated coupled proton–electron transfer. Org. Biomol. Chem..

[83] Iordănescu O.A., Băla M., Gligor-(Pane) D., Zippenfening S.E., Cugerean M.I., Petroman M.I., Hădărugă D.I., Hădărugă N.G., Riviş M. (2021). A DPPH· Kinetic Approach on the Antioxidant Activity of Various Parts and Ripening Levels of Papaya (*Carica papaya* L.) Ethanolic Extracts. Plants.

[84] Iordănescu O.A., Băla M., Iuga A.C., Gligor-(Pane) D., Dascălu I., Bujancă G.S., David I., Hădărugă N.G., Hădărugă D.I. (2021). Antioxidant activity and discrimination of organic apples (*Malus domestica* Borkh.) cultivated in the western region of Romania: A DPPH· kinetics–PCA approach. Plants.

[85] Jabbari M., Jabbari A. (2016). Antioxidant potential and DPPH radical scavenging kinetics of water-insoluble flavonoid naringenin in aqueous solution of micelles. Colloids Surf. A Physicochem. Eng. Asp..

[86] Pannala A.S., Chan T.S., O’Brien P.J., Rice-Evans C.A. (2001). Flavonoid B-Ring Chemistry and Antioxidant Activity: Fast Reaction Kinetics. Biochem. Biophys. Res. Commun..

[87] Campos A.M., Lissi E.A. (1997). Kinetics of the Reaction between 2,2′-Azinobis(3-Ethylbenzothiazoline-6-Sulfonic Acid) (ABTS) Derived Radical Cations and Phenols. Int. J. Chem. Kinet..

[88] Henriquez C., Aliaga C., Lissi E. (2004). Kinetics profiles in the reaction of ABTS derived radicals with simple phenols and polyphenols. J. Chil. Chem. Soc..

[89] Pérez-Jiménez J., Saura-Calixto F. (2008). Anti-oxidant capacity of dietary polyphenols determined by ABTS assay: A kinetic expression of the results. Int. J. Food Sci. Technol..

[90] Hădărugă N.-G., Popescu G., Gligor-(Pane) D., Mitroi C.L., Stanciu S.M., Hădărugă D.I. (2023). Discrimination of β-cyclodextrin/hazelnut (*Corylus avellana* L.) oil/flavonoid glycoside and flavonolignan ternary complexes by Fourier-transform infrared spectroscopy coupled with principal component analysis. Beilstein J. Org. Chem..

[91] Horablaga A., Şibu-(Ciobanu) A., Megyesi C.I., Gligor-(Pane) D., Bujancă G.S., Velciov A.B., Morariu F.E., Hădărugă D.I., Mişcă C.D., Hădărugă N.G. (2023). Estimation of the Controlled Release of Antioxidants from β-Cyclodextrin/Chamomile (*Matricaria chamomilla* L.) or Milk Thistle (*Silybum marianum* L.), Asteraceae, Hydrophilic Extract Complexes through the Fast and Cheap Spectrophotometric Technique. Plants.

[92] Cai R., Yuan Y., Cui L., Wang Z., Yue T. (2018). Cyclodextrin-assisted extraction of phenolic compounds: Current research and future prospects. Trends Food Sci. Technol..

[93] You G.-J., Sun L.-L., Cao X.-X., Li H.-H., Wang M., Liu Y.-N., Ren X.-L. (2018). Comprehensive evaluation of solubilization of flavonoids by various cyclodextrins using high performance liquid chromatography and chemometry. LWT—Food Sci. Technol..

[94] Lucas-Abellán C., Pérez-Abril M., Castillo J., Serrano A., Mercader M.T., Fortea M.I., Gabaldón J.A., Núñez-Delicado E. (2019). Effect of temperature, pH, β- and HP-β-cds on the solubility and stability of flavanones: Naringenin and hesperetin. LWT—Food Sci. Technol..

[95] Ratnasooriya C.C., Rupasinghe H.P.V. (2012). Extraction of phenolic compounds from grapes and their pomace using β-cyclodextrin. Food Chem..

[96] Krstić L., Jarho P., Ruponen M., Urtti A., González-García M.J., Diebold Y. (2022). Improved ocular delivery of quercetin and resveratrol: A comparative study between binary and ternary cyclodextrin complexes. Int. J. Pharm..

[97] Nguyen T.A., Liu B., Zhao J., Thomas D.S., Hook J.M. (2013). An investigation into the supramolecular structure, solubility, stability and antioxidant activity of rutin/cyclodextrin inclusion complex. Food Chem..

[98] Pinhoa E., Grootveld M., Soares G., Henriques M. (2014). Cyclodextrins as encapsulation agents for plant bioactive compounds. Carbohydr. Polym..

[99] Xiong Y., Chang M., Shi Z.-W., Li Y.-Y., An S.-Y., Ren D.-F. (2025). Encapsulation of rose anthocyanins with β-cyclodextrin for enhanced stability: Preparation, characterization, and its application in rose juice. Food Biosci..

[100] Mourtzinos I., Makris D.P., Yannakopoulou K., Kalogeropoulos N., Michali I., Karathanos V.T. (2008). Thermal Stability of Anthocyanin Extract of *Hibiscus sabdariffa* L. in the Presence of β-Cyclodextrin. J. Agric. Food Chem..

[101] Xie J., Xu Y., Shishir M.R.I., Zheng X., Chen W. (2019). Green extraction of mulberry anthocyanin with improved stability using β-cyclodextrin. J. Sci. Food Agric..

[102] Peron D.V., Fraga S., Antelo F. (2017). Thermal degradation kinetics of anthocyanins extracted from juçara (*Euterpe edulis* Martius) and ‘‘Italia” grapes (*Vitis vinifera* L.), and the effect of heating on the antioxidant capacity. Food Chem..

[103] Kim A.-N., Kim H.-J., Chun J., Heo H.J., Kerr W.L., Choi S.-G. (2018). Degradation kinetics of phenolic content and antioxidant activity of hardy kiwifruit (*Actinidia arguta*) puree at different storage temperatures. LWT—Food Sci. Technol..

[104] Rodríguez-Ramírez J., Méndez-Lagunas L.L., López-Ortiz A., Muñiz-Becerá S., Nair K. (2021). Solar drying of strawberry using polycarbonate with UV protection and polyethylene covers: Influence on anthocyanin and total phenolic content. Sol. Energy.

[105] Méndez-Lagunas L., Rodríguez-Ramírez J., Cruz-Gracida M., Sandoval-Torres S., Barriada-Bernal G. (2017). Convective drying kinetics of strawberry (*Fragaria ananassa*): Effects on antioxidant activity, anthocyanins and total phenolic content. Food Chem..

[106] Méndez-Lagunas L.L., Rodríguez-Ramírez J., Sandoval-Torres S., Barragán-Iglesias J., López-Ortíz A. (2025). Strawberry fluidized bed drying. Antiadhesion pretreatments and their effect on bioactive compounds. Appl. Food Res..

[107] Stojiljković D., Arsić I., Tadić V. (2016). Extracts of wild apple fruit (*Malus sylvestris* (L.) Mill., Rosaceae) as asource of antioxidant substances for use in production ofnutraceuticals and cosmeceuticals. Ind. Crops Prod..

[108] Veggi P.C., Santos D.T., Meireles M.A.A. (2011). Anthocyanin extraction from Jabuticaba (*Myrciaria cauliflora*) skins by different techniques: Economic evaluation. Procedia Food Sci..

[109] Zhang Z., Kou X., Fugal K., McLaughlin J. (2004). Comparison of HPLC Methods for Determination of Anthocyanins and Anthocyanidins in Bilberry Extracts. J. Agric. Food Chem..

[110] Ghose A.K., Pritchett A., Crippen G.M. (1988). Atomic Physicochemical Parameters for Three Dimensional Structure Directed Quantitative Structure–Activity Relationships III: Modeling Hydrophobic Interactions. J. Comput. Chem..

[111] Viswanadhan V.N., Ghose A.K., Reyankar G.R., Robins R.K. (1989). Atomic Physicochemical Parameters for Three Dimensional Structure Directed Quantitative Structure–Activity Relationships. 4. Additional Parameters for Hydrophobic and Dispersive Interactions and Their Application for an Automated Superposition of Certain Naturally Occurring Nucleoside Antibiotics. J. Chem. Inf. Comput. Sci..

