# Supplementary material for: Influence of β-Cyclodextrin on the Overall Antioxidant Activity and DPPH· Reaction Kinetics of Fresh Raspberry (Rubus idaeus L.) and Dehydrated Strawberry (Fragaria × ananassa Duch.) Extracts
Source: Plants (Basel). 2026 Jan 4;15(1):152. doi: 10.3390/plants15010152 (PMC12787975; doi:10.3390/plants15010152)
Supplement: Supplementary file 1 [file plants-15-00152-s001.zip › plants-4060537-supplementary.pdf]

plants

Article

Supplementary Material

Influence of β-Cyclodextrin on the Overall Antioxidant Activity and DPPH· Reaction Kinetics of Fresh Raspberry (*Rubus idaeus* L.) and Dehydrated Strawberry (*Fragaria × ananassa* Duch.) Extracts

Marinela Fiţoiu (Voin) [1], Anamaria Pop (Mateuţ) [1], Elena Vladu [1], Roxana Poja [1], Lavinia-Alexandra Toporîşte [2], Carina Elena Molnar [2], Mărioara Drugă [3], Gabriel Stelian Bujancă [4], Ioan David [3], Adina Horablaga [5], Nicoleta-Gabriela Hădărugă [1,3,*], and Daniel-Ioan Hădărugă [1,2,*]

[1] Doctoral School "Engineering of Vegetable and Animal Resources", University of Life Sciences "King Mihai I" from Timişoara, Calea Aradului 119, 300645-Timişoara, Romania
[2] Department of Applied Chemistry, Organic and Natural Compounds Engineering, Polytechnic University of Timişoara, Vasile Pârvan Bd. 6, 300223 – Timişoara, Romania
[3] Department of Food Science, University of Life Sciences "King Mihai I" from Timişoara, Calea Aradului 119, 300645 – Timişoara, Romania
[4] Department of Food Control and Expertise, University of Life Sciences "King Mihai I" from Timişoara, Calea Aradului 119, 300645 – Timişoara, Romania
[5] Department of Sustainable Development and Environmental Engineering, University of Life Sciences "King Mihai I" from Timişoara, Calea Aradului 119, 300645-Timişoara, Romania
* Correspondence: nicoletahadaruga@usvt.ro; nico_hadaruga@yahoo.com (N.-G.H.); daniel.hadaruga@upt.ro (D.-I.H.)

1. HPLC calibration curves for cyanidin 3-*O*-glucoside and cyanidin standard solutions

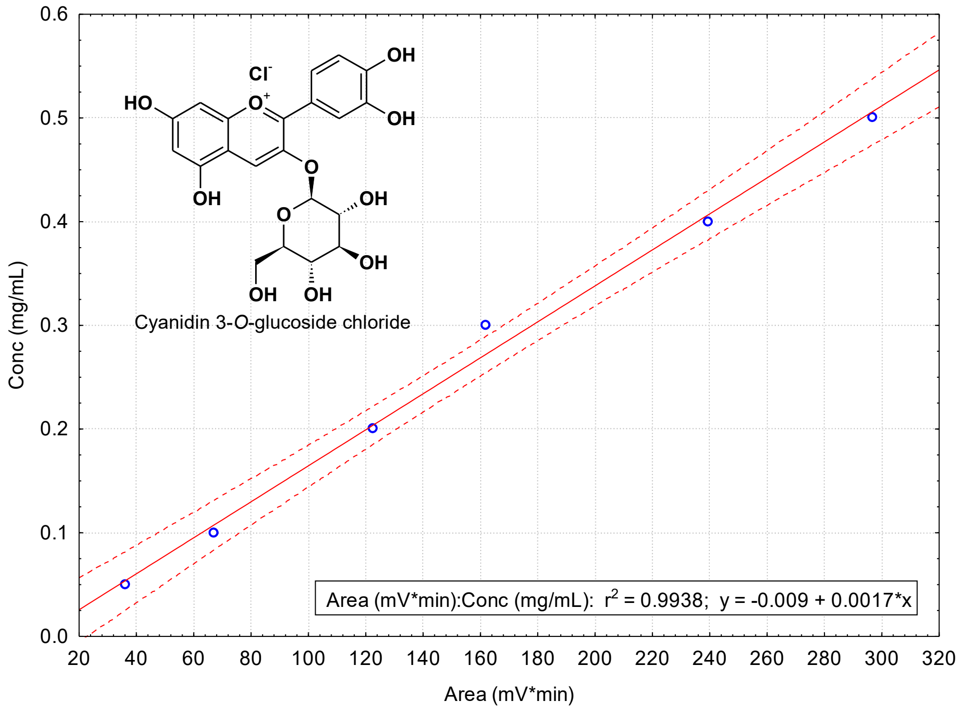

Figure S1. Concentration (mg/mL) versus HPLC peak area (mV·min) calibration curve for the cyanidin 3-*O*-glucoside standard solutions in the range of 0.05-0.50 mg/mL.

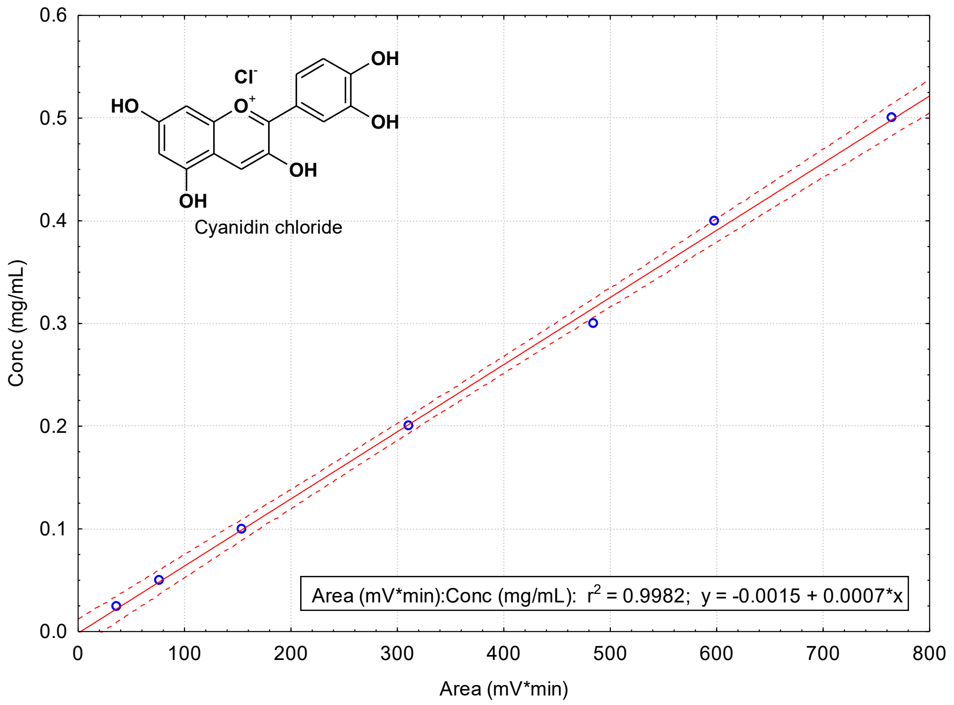

Figure S2. Concentration (mg/mL) versus HPLC peak area (mV·min) calibration curve for the cyanidin standard solutions in the range of 0.025-0.50 mg/mL.

2. UV-Vis spectra for the selected 2,2-diphenyl-1-pycrylhydrazyl (DPPH·) and cyanidin 3-*O*-glucoside (Cy3G) standard solutions

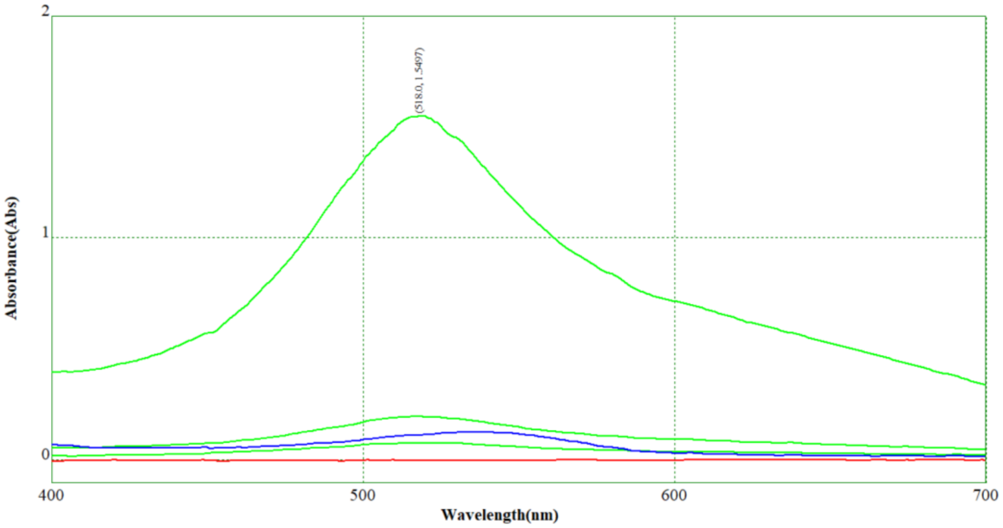

Figure S3. Comparison between the UV-Vis spectra (range of 400-700 nm) of the cyanidin 3-*O*-glucoside (Cy3G) standard solution (e.g., 0.06 mM – blue) and the standard DPPH· solutions (e.g. from bottom to top: 0.01, 0.02 and 0.16 mM, which is the working concentration at the start of the measurements – green). The Cy3G standard solution has a maximum absorbance in the range of the high diluted DPPH· solutions of 0.01 and 0.02 mM. However, the Cy3G working concentration at the start of the measurement is four times lower (0.015 mM). The spectra for the blank sample (ethanol) is in red.

3. UV-Vis spectra and Concentration versus Absorbance (@517 nm) calibration curve for 2,2-diphenyl-1-pycrylhydrazyl (DPPH·) standard solutions

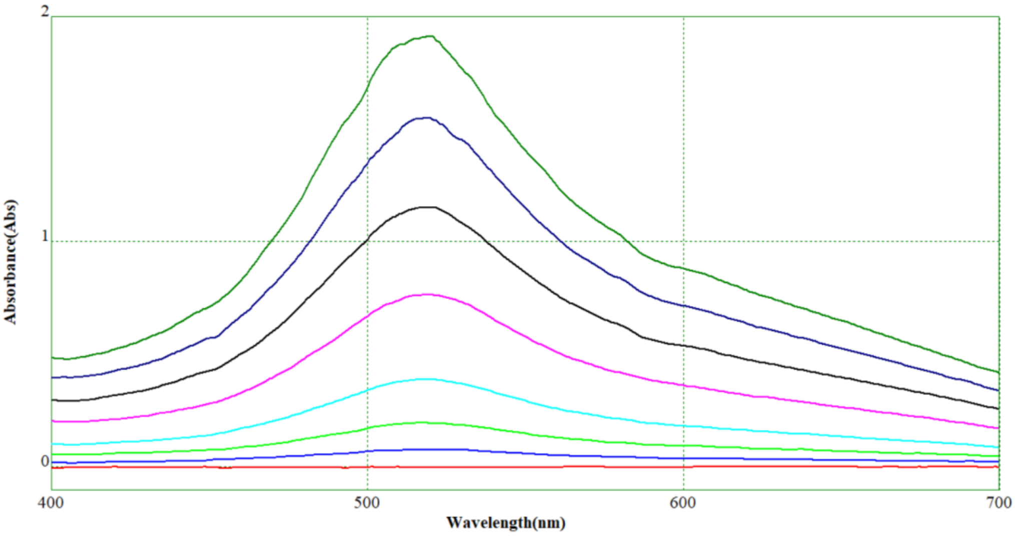

Figure S4. UV-Vis spectra (region 400-700 nm) of the DPPH· standard solutions (from bottom to top, concentrations from 0.01, 0.02, 0.04, 0.08, 0.12, 0.16 and 0.20 mM). The spectra for the blank sample (ethanol) is in red.

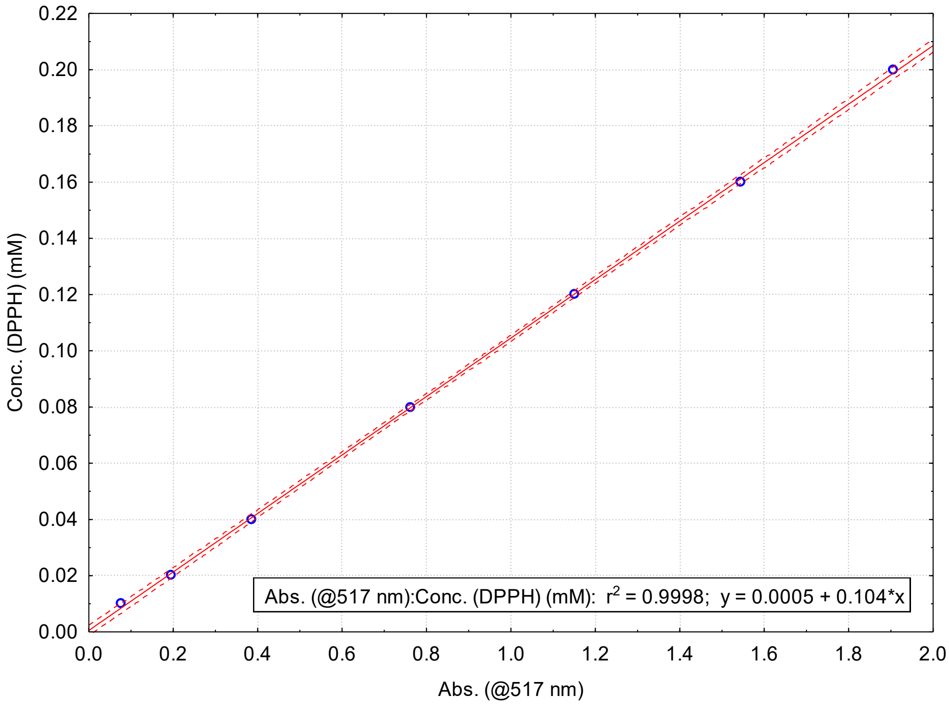

Figure S5. Concentration (mM) versus Absorbance (@517 nm) calibration curve for the DPPH· standard solutions in the range of 0.01-0.20 mM.

4. UV-Vis monitoring for cyanidin 3-*O*-glucoside, DPPH· and β-cyclodextrin mixtures

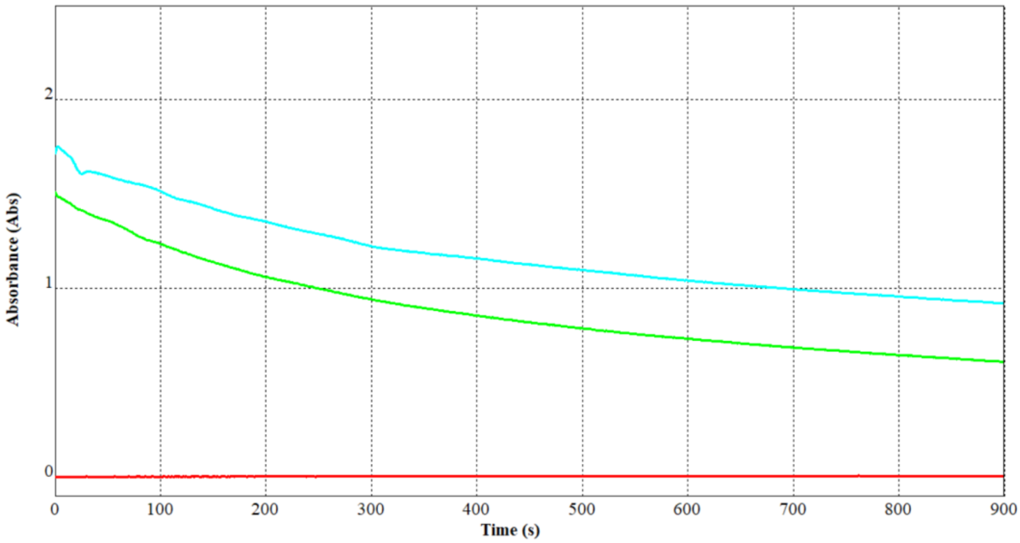

Figure S6. UV-Vis monitoring (at 517 nm) of the 0.4 mM cyanidin 3-*O*-glucoside and 1 mM DPPH· solutions in ethanol, at a volume ratio of 1:1:4 (duplicate samples; blank ethanol is in red).

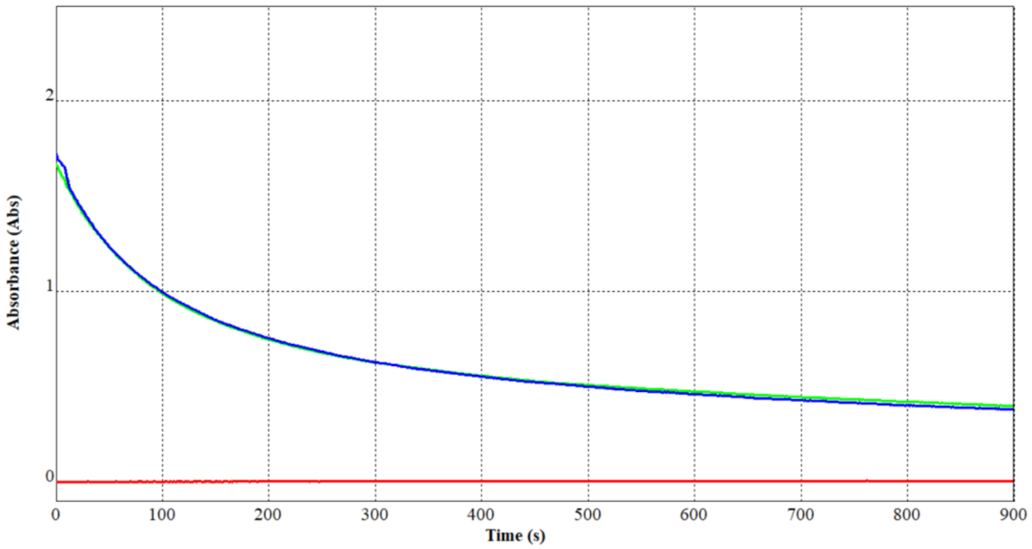

Figure S7. UV-Vis monitoring (at 517 nm) of the 0.4 mM cyanidin 3-*O*-glucoside, 1 mM DPPH· and 1 mM β-cyclodextrin (in water) solutions, mixed with ethanol at a volume ratio of 1:1:1:3 (duplicate samples; blank ethanol is in red).

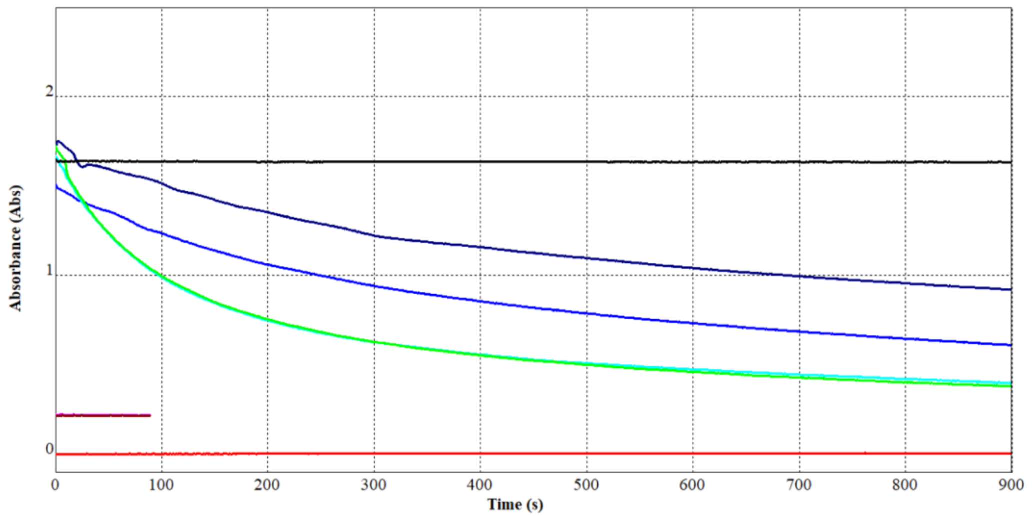

Figure S8. Superimposed Absorbance versus Time plots from the UV-Vis monitoring (at 517 nm) of the 0.4 mM cyanidin 3-*O*-glucoside and 1 mM DPPH· solutions in ethanol at 1:1:4 volume ratio (duplicate samples; blue colors) and for the 0.4 mM cyanidin 3-*O*-glucoside, 1 mM DPPH· and 1 mM β-cyclodextrin (in water) solutions, mixed with ethanol at a volume ratio of 1:1:1:3 (duplicate samples; green colors) (blank ethanol is in red, blank cyanidin 3-*O*-glucoside solution is in purple and blank DPPH· solution is in black).

5. Antioxidant activity of the fresh and dehydrated strawberry extracts

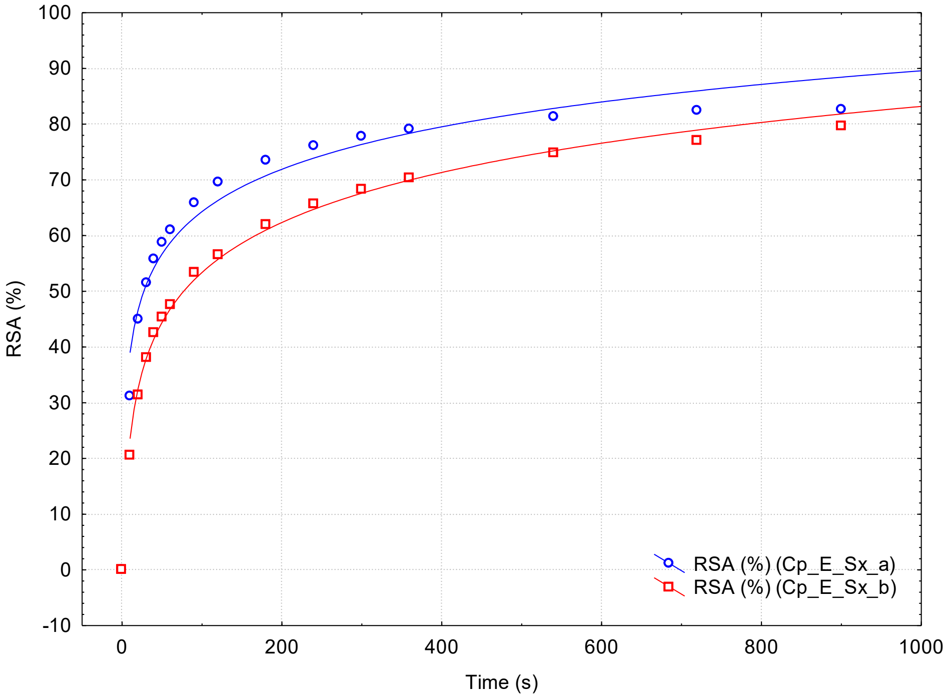

Figure S9. Antioxidant activity of the fresh strawberry extracts, expressed as the variation in the Radical Scavenging Activity (RSA, %) in Time (s) for 15 min, as well as the logarithmic correlations (duplicate samples).

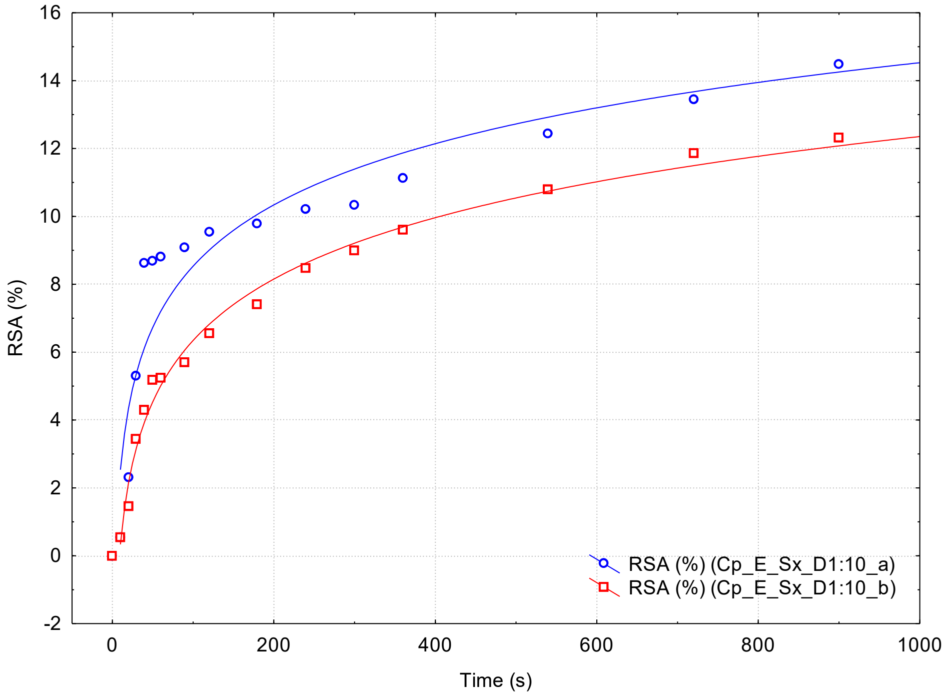

Figure S10. Antioxidant activity of the fresh strawberry extracts (1:10 dilutions), expressed as the variation in the Radical Scavenging Activity (RSA, %) in Time (s) for 15 min, as well as the logarithmic correlations (duplicate samples).

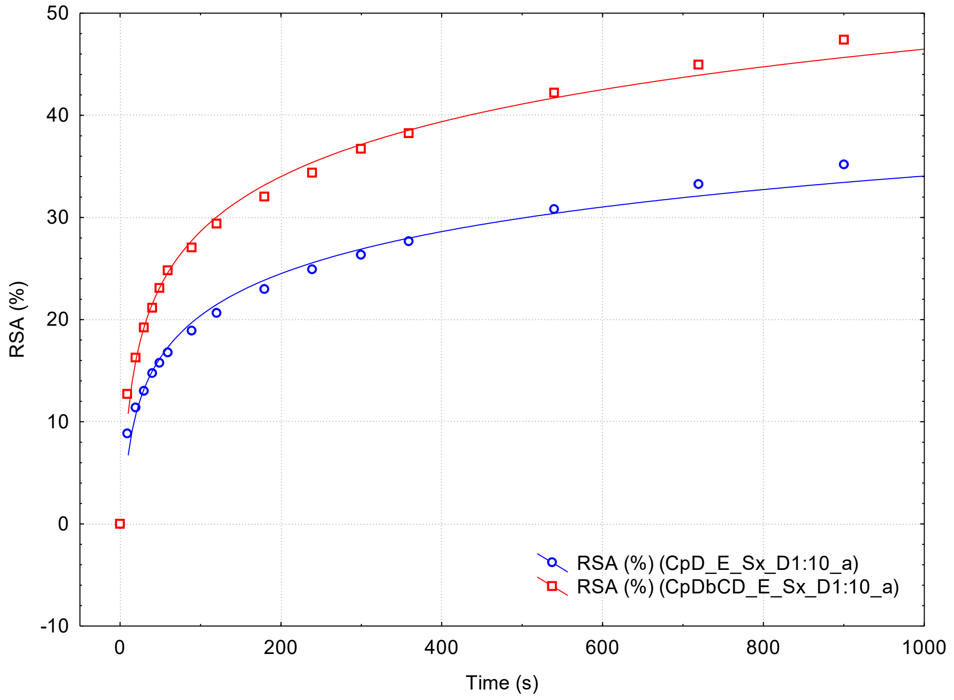

Figure S11. Antioxidant activity of the extracts obtained from dehydrated strawberries, without (blue) and with β-cyclodextrin (red) as additive (1:10 dilutions). Antioxidant activity is expressed as the variation in the Radical Scavenging Activity (RSA, %) in Time (s) for 15 min. Logarithmic correlations are also provided.

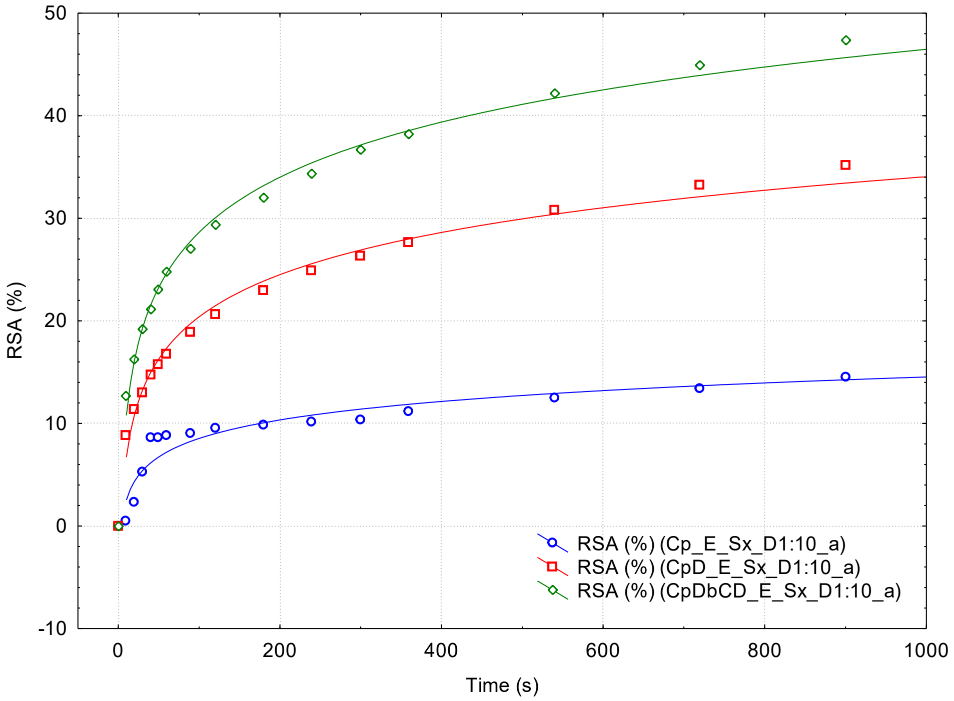

Figure S12. Antioxidant activity of the extracts obtained from fresh (blue) and dehydrated strawberries, without (red) and with β-cyclodextrin (green) as additive (1:10 dilutions). Antioxidant activity is expressed as the variation in the Radical Scavenging Activity (RSA, %) in Time (s) for 15 min. Logarithmic correlations are also provided.

6. Antioxidant activity of the fresh raspberry extracts (β-cyclodextrin assisted or not assisted during spectrophotometric monitoring)

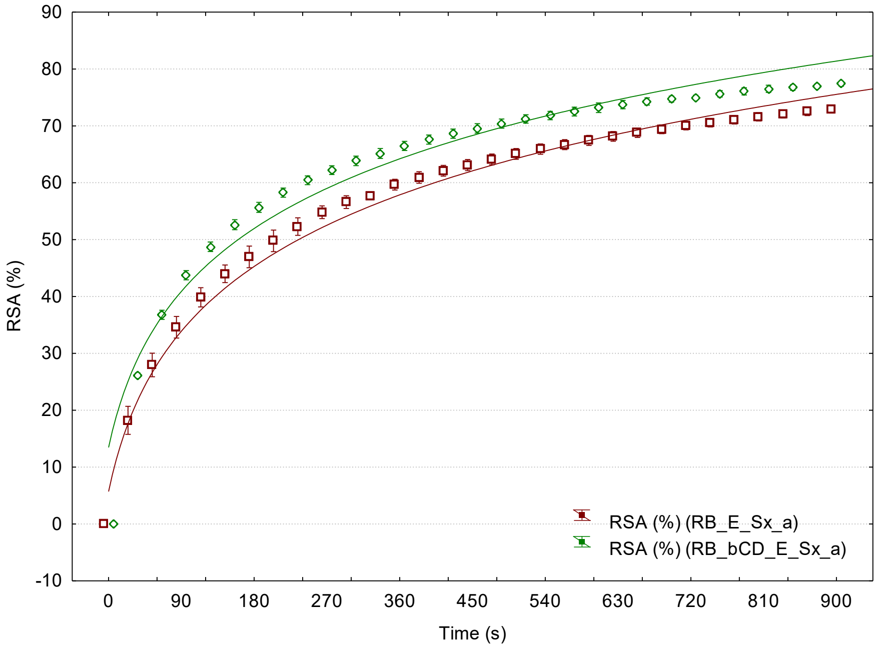

Figure S13. Antioxidant activity of the extracts obtained from fresh raspberries, β-cyclodextrin assisted (green diamond) or not assisted (brown square) during the spectrophotometric monitoring (duplicate samples). Antioxidant activity is expressed as the variation in the Radical Scavenging Activity (RSA, %) in Time (s) for 15 min. For multiple determinations, Whisker type plots as mean ± 0.95·standard error were used. Logarithmic correlations are also provided.

7. Antioxidant activity of the standard 0.4 mM cyanidin 3-*O*-glucoside solutions (β-cyclodextrin assisted or not assisted during spectrophotometric monitoring)

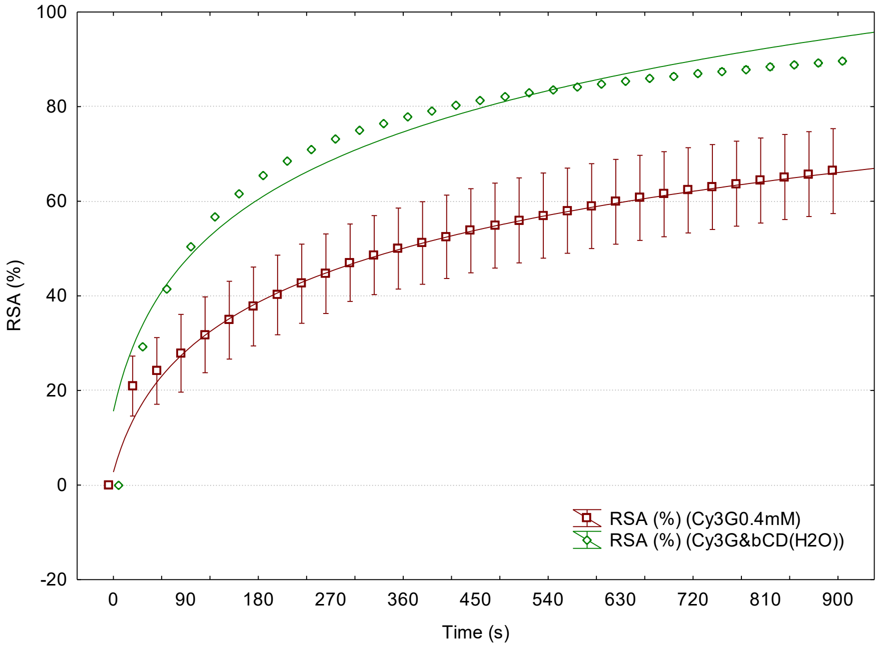

Figure S14. Antioxidant activity of the standard 0.4 mM cyanidin 3-*O*-glucoside, β-cyclodextrin assisted (green diamond) or not assisted (brown square) during the spectrophotometric monitoring (duplicate samples). Antioxidant activity is expressed as the variation in the Radical Scavenging Activity (RSA, %) in Time (s) for 15 min. For multiple determinations, Whisker type plots as mean ± 0.95·standard error were used. Logarithmic correlations are also provided.

8. Kinetic parameters for the reaction of DPPH· with antioxidant compounds from the fresh and dehydrated strawberry extracts

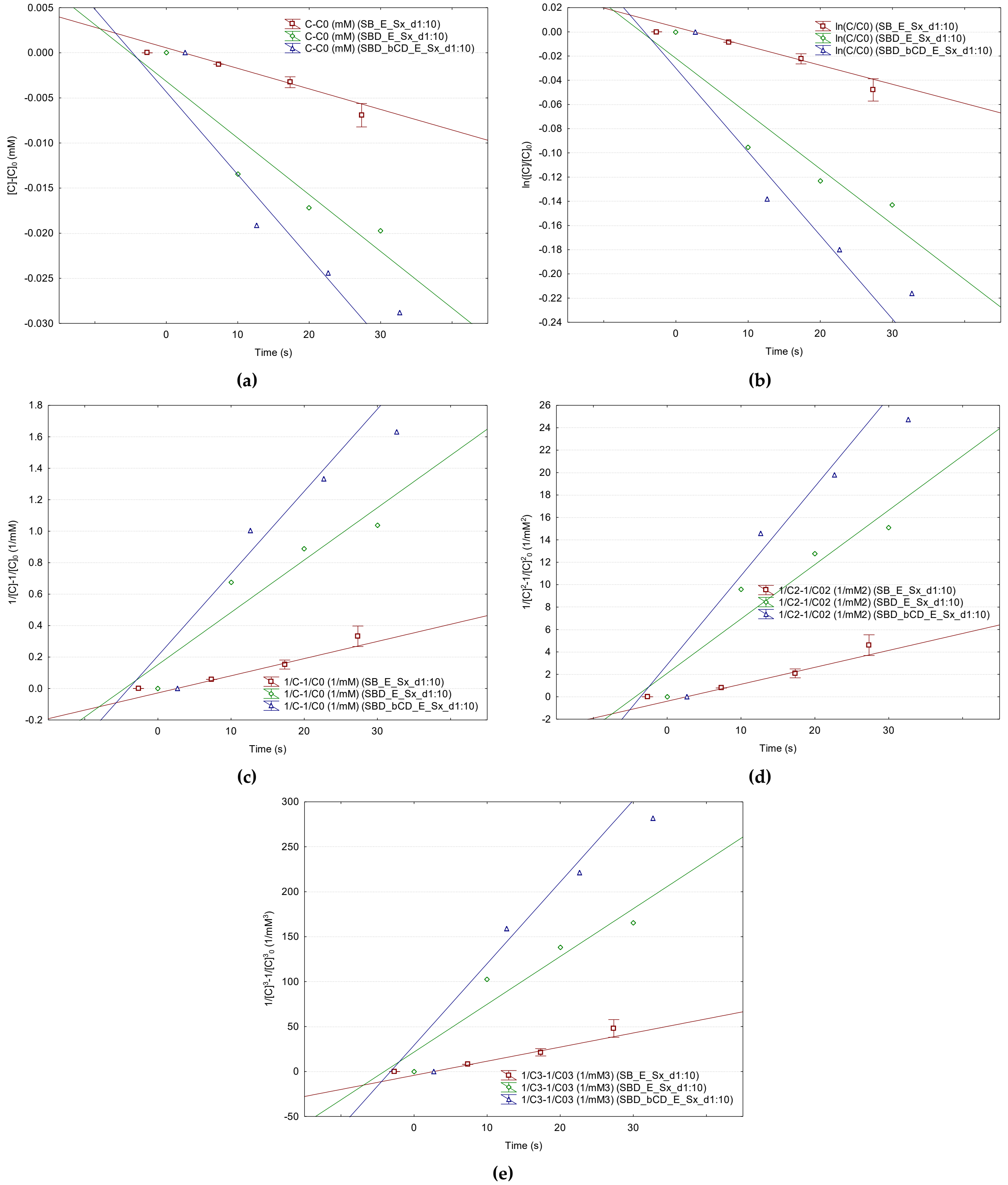

Figure S15. Integrated rate law representation for the zeroth–fourth-order models for the "fast" DPPH· reaction with antioxidant compounds from the extracts obtained from the fresh and dehydrated strawberries (without or with β-cyclodextrin as additive; 1:10 diluted extracts). For duplicate samples, Whisker type plots as mean ± 0.95·standard error were used.

Table S1. Kinetic and statistic parameters corresponding to the zeroth–fourth-order models for the "fast" DPPH· reaction with antioxidant compounds from the 1:10 diluted extracts obtained from the fresh and dehydrated strawberries (without or with β-cyclodextrin as additive). For 1:10 diluted samples, k values with different superscript capital letters are significantly different, according to Tukey's HSD test ($p < 0.01$; on the same raw). For 1:10 diluted samples, $t_{1/2}$ values with different superscript lowercase letters are significantly different, according to Tukey's HSD test ($p < 0.05$; on the same raw).

**Parameter**	***SB_d1:10***	***SBD_d1:10***	***SBD_bCD_d1:10***
Rate constant, k_0 (mM/s)	0.00023^{A}	0.00063^{B}	0.00092^{C}
Half-life, $t_{1/2(0)}$ (s)	321.7^{a}	117.5^{a}	80.4^{b}
Determination coefficient, $r^2_{(0)}$	0.946	0.855	0.87
Rate constant, k_1 (1/s)	0.0016^{A}	0.0046^{B}	0.0069^{C}
Half-life, $t_{1/2(1)}$ (s)	433.2^{a}	150.7^{a}	100.5^{b}
Determination coefficient, $r^2_{(1)}$	0.943	0.866	0.888
Rate constant, k_2 (1/(mM·s))	0.011^{A}	0.033^{B}	0.052^{C}
Half-life, $t_{1/2(2)}$ (s)	614.3^{a}	204.8^{a}	129.9^{b}
Determination coefficient, $r^2_{(2)}$	0.940	0.878	0.904
Rate constant, k_3 (1/(mM²·s))	0.151^{A}	0.485^{B}	0.794^{C}
Half-life, $t_{1/2(3)}$ (s)	453.5^{a}	141.2^{a}	86.2^{b}
Determination coefficient, $r^2_{(3)}$	**0.936**	**0.889**	**0.920**
Rate constant, k_4 (1/(mM³·s))	1.57^{A}	5.315^{B}	9.08^{C}
Half-life, $t_{1/2(4)}$ (s)	458.5^{a}	135.4^{a}	79.3^{b}
Determination coefficient, $r^2_{(4)}$	**0.933**	**0.900**	**0.935**

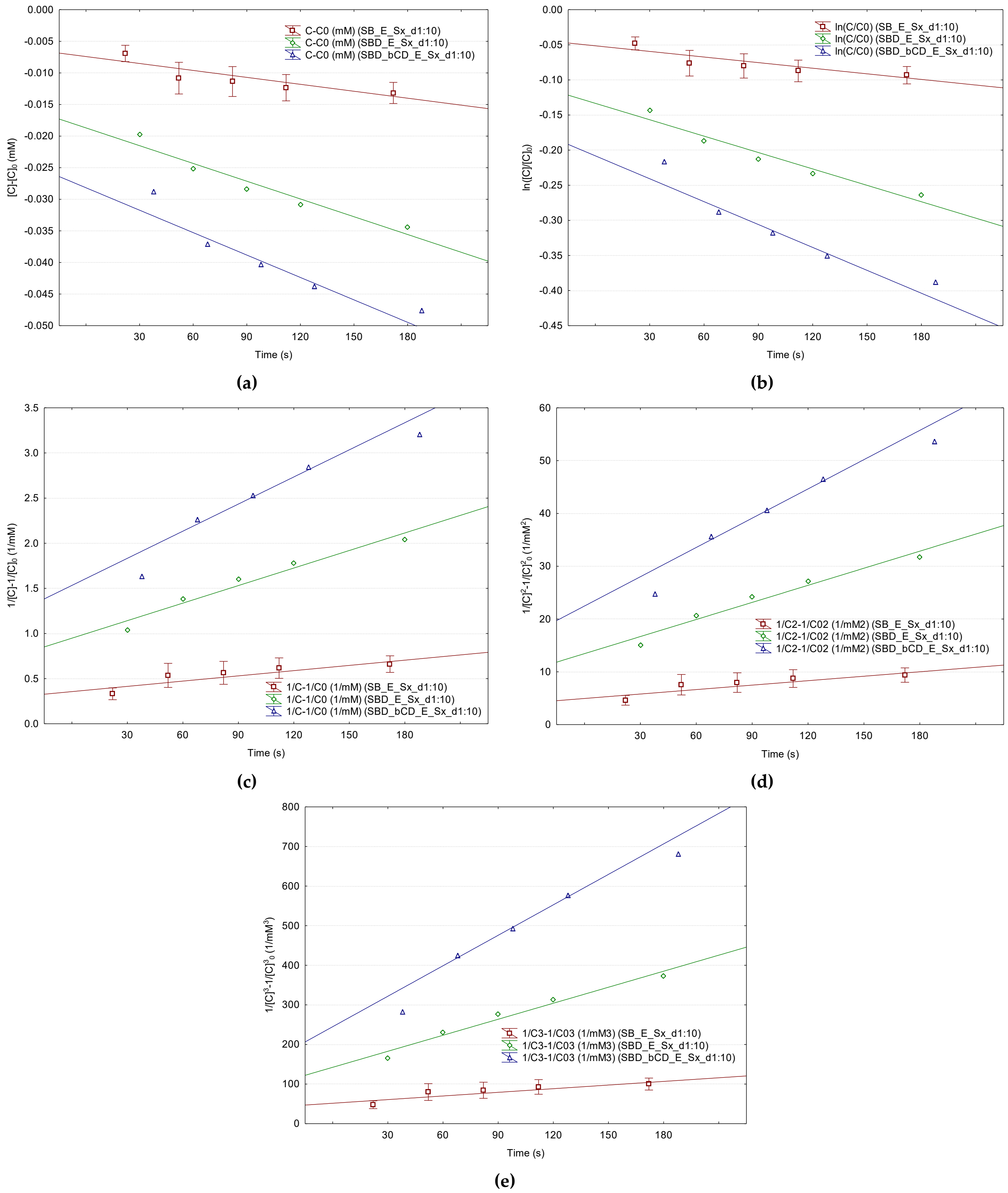

Figure S16. Integrated rate law representation for the zeroth–fourth-order models for the "slow" DPPH· reaction with antioxidant compounds from the extracts obtained from the fresh and dehydrated strawberries (without or with β-cyclodextrin as additive; 1:10 diluted extracts). For duplicate samples, Whisker type plots as mean ± 0.95·standard error were used.

Table S2. Kinetic and statistic parameters corresponding to the zeroth–fourth-order models for the "slow" DPPH· reaction with antioxidant compounds from the 1:10 diluted extracts obtained from the fresh and dehydrated strawberries (without or with β-cyclodextrin as additive). For 1:10 diluted samples, k values with different superscript capital letters are significantly different, according to Tukey's HSD test ($p < 0.001$; on the same raw). For 1:10 diluted samples, $t_{1/2}$ values with different superscript lowercase letters are significantly different, according to Tukey's HSD test ($p < 0.01$; on the same raw).

**Parameter**	***SB_d1:10***	***SBD_d1:10***	***SBD_bCD_d1:10***
Rate constant, k_0 (mM/s)	0.000031 ^{A}	0.000094 ^{B}	0.000121 ^{C}
Half-life, $t_{1/2(0)}$ (s)	2387.1 ^{a}	787.2 ^{b}	611.6 ^{b}
Determination coefficient, $r^2_{(0)}$	0.695	0.949	0.922
Rate constant, k_1 (1/s)	0.00023 ^{A}	0.00078 ^{B}	0.0011 ^{C}
Half-life, $t_{1/2(1)}$ (s)	3013.7 ^{a}	888.7 ^{b}	630.1 ^{b}
Determination coefficient, $r^2_{(1)}$	0.698	0.957	0.935
Rate constant, k_2 (1/(mM·s))	0.00165 ^{A}	0.0065 ^{B}	0.0102 ^{C}
Half-life, $t_{1/2(2)}$ (s)	4095.0 ^{a}	1039.5 ^{b}	662.4 ^{b}
Determination coefficient, $r^2_{(2)}$	0.701	0.964	0.946
Rate constant, k_3 (1/(mM^2·s))	0.024 ^{A}	0.108 ^{B}	0.188 ^{C}
Half-life, $t_{1/2(3)}$ (s)	2853.4 ^{a}	634.1 ^{b}	364.3 ^{b}
Determination coefficient, $r^2_{(3)}$	**0.704**	**0.970**	**0.957**
Rate constant, k_4 (1/(mM^3·s))	0.262 ^{A}	1.351 ^{B}	2.61 ^{C}
Half-life, $t_{1/2(4)}$ (s)	2747.2 ^{a}	532.8 ^{b}	275.8 ^{b}
Determination coefficient, $r^2_{(4)}$	**0.708**	**0.976**	**0.966**

9. Kinetic parameters for the reaction of DPPH· with antioxidant compounds from the fresh raspberry extracts (β-cyclodextrin assisted or not assisted during spectrophotometric monitoring)

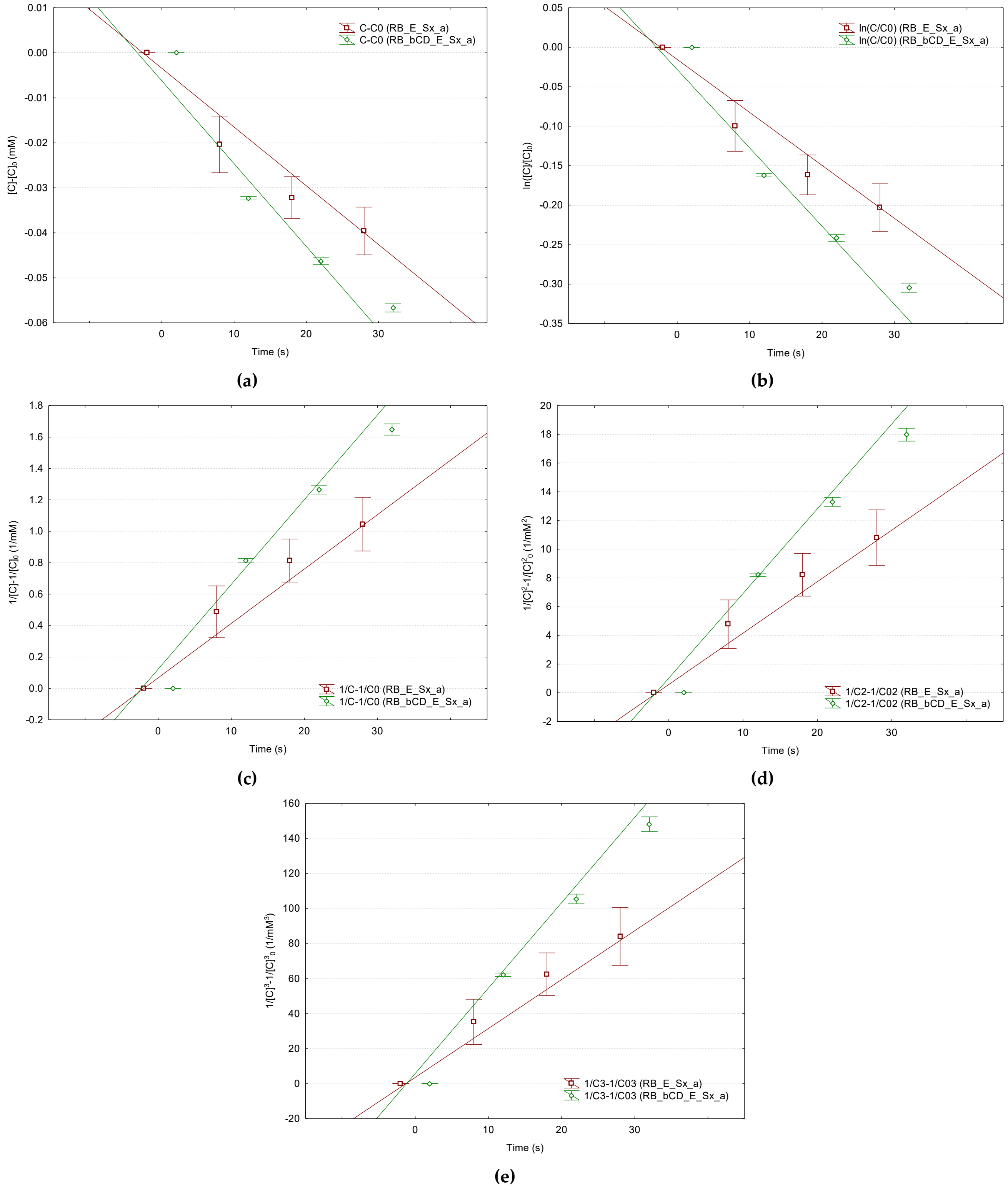

Figure S17. Integrated rate law representation for the zeroth–fourth-order models for the "fast" DPPH· reaction with antioxidant compounds from the extracts obtained from the fresh raspberries (without or with β-cyclodextrin as additive during the spectrophotometric monitoring). For duplicate samples, Whisker type plots as mean ± 0.95·standard error were used.

Table S3. Kinetic and statistic parameters corresponding to the zeroth–fourth-order models for the "fast" DPPH· reaction with antioxidant compounds from the extracts obtained from the fresh raspberries (without or with β-cyclodextrin as additive during the spectrophotometric monitoring). On the same row, k values with different superscript capital letters are significantly different, according to Tukey's HSD test ($p < 0.10$). On the same row, $t_{1/2}$ values with different superscript lowercase letters are significantly different, according to Tukey's HSD test ($p < 0.15$).

**Parameter**	***RB***	***RB_bCD***
Rate constant, k_0 (mM/s)	0.0013^{A}	0.0018^{B}
Half-life, $t_{1/2(0)}$ (s)	83.1^{a}	60.0^{b}
Determination coefficient, $r^2_{(0)}$	0.952	0.928
Rate constant, k_1 (1/s)	0.0067^{A}	0.0099^{B}
Half-life, $t_{1/2(1)}$ (s)	103.5^{a}	70.0^{b}
Determination coefficient, $r^2_{(1)}$	0.963	0.949
Rate constant, k_2 (1/(mM·s))	0.0346^{A}	0.0539^{B}
Half-life, $t_{1/2(2)}$ (s)	133.2^{a}	85.9^{b}
Determination coefficient, $r^2_{(2)}$	0.973	0.966
Rate constant, k_3 (1/(mM^2·s))	0.358^{A}	0.590^{B}
Half-life, $t_{1/2(3)}$ (s)	89.8^{a}	54.5^{b}
Determination coefficient, $r^2_{(3)}$	**0.981**	**0.980**
Rate constant, k_4 (1/(mM^3·s))	2.795^{A}	4.879^{B}
Half-life, $t_{1/2(4)}$ (s)	82.8^{a}	47.5^{b}
Determination coefficient, $r^2_{(4)}$	**0.988**	**0.991**

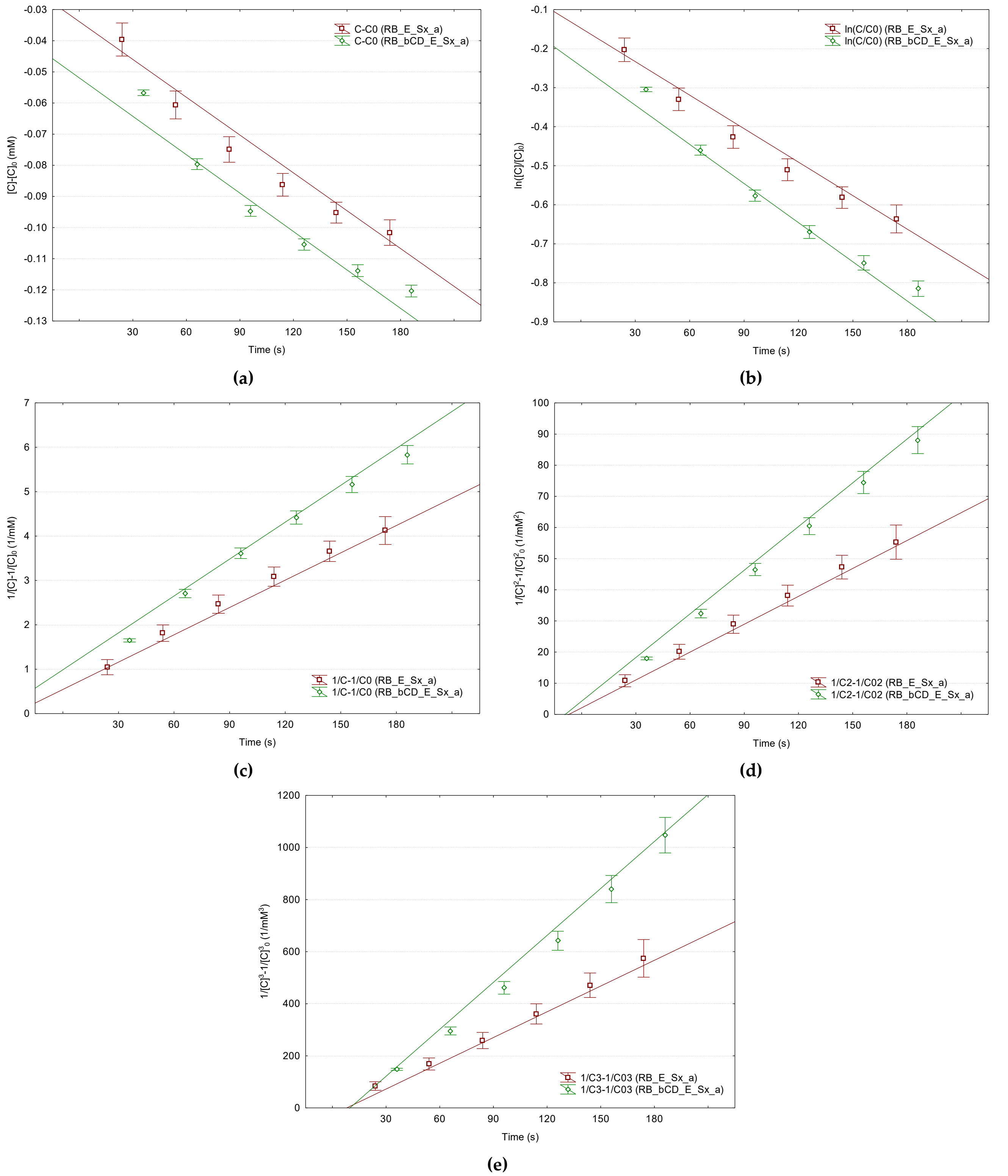

Figure S18. Integrated rate law representation for the zeroth–fourth-order models for the "slow" DPPH· reaction with antioxidant compounds from the extracts obtained from the fresh raspberries (without or with β-cyclodextrin as additive during the spectrophotometric monitoring). For duplicate samples, Whisker type plots as mean ± 0.95·standard error were used.

Table S4. Kinetic and statistic parameters corresponding to the zeroth–fourth-order models for the "slow" DPPH· reaction with antioxidant compounds from the extracts obtained from the fresh raspberries (without or with β-cyclodextrin as additive during the spectrophotometric monitoring). On the same row, k values with different superscript capital letters are significantly different, according to Tukey's HSD test ($p < 0.05$). On the same row, $t_{1/2}$ values with different superscript lowercase letters are significantly different, according to Tukey's HSD test ($p < 0.05$).

**Parameter**	***RB***	***RB_bCD***
Rate constant, k_0 (mM/s)	0.00041^{A}	0.00042^{A}
Half-life, $t_{1/2(0)}$ (s)	263.4^{a}	257.1^{a}
Determination coefficient, $r^2_{(0)}$	0.969	0.960
Rate constant, k_1 (1/s)	0.0029^{A}	0.0034^{B}
Half-life, $t_{1/2(1)}$ (s)	239.0^{a}	203.9^{b}
Determination coefficient, $r^2_{(1)}$	0.985	0.982
Rate constant, k_2 (1/(mM·s))	0.021^{A}	0.029^{B}
Half-life, $t_{1/2(2)}$ (s)	220.5^{a}	159.6^{b}
Determination coefficient, $r^2_{(2)}$	0.995	0.995
Rate constant, k_3 (1/(mM^2·s))	0.298^{A}	0.467^{B}
Half-life, $t_{1/2(3)}$ (s)	107.9^{a}	68.80^{b}
Determination coefficient, $r^2_{(3)}$	**1.000**	**1.000**
Rate constant, k_4 (1/(mM^3·s))	3.277^{A}	5.966^{B}
Half-life, $t_{1/2(4)}$ (s)	70.70^{a}	38.80^{a}
Determination coefficient, $r^2_{(4)}$	**0.998**	**0.997**

10. Kinetic parameters for the reaction of DPPH· with the standard cyanidin 3-*O*-glucoside (β-cyclodextrin assisted or not assisted during spectrophotometric monitoring)

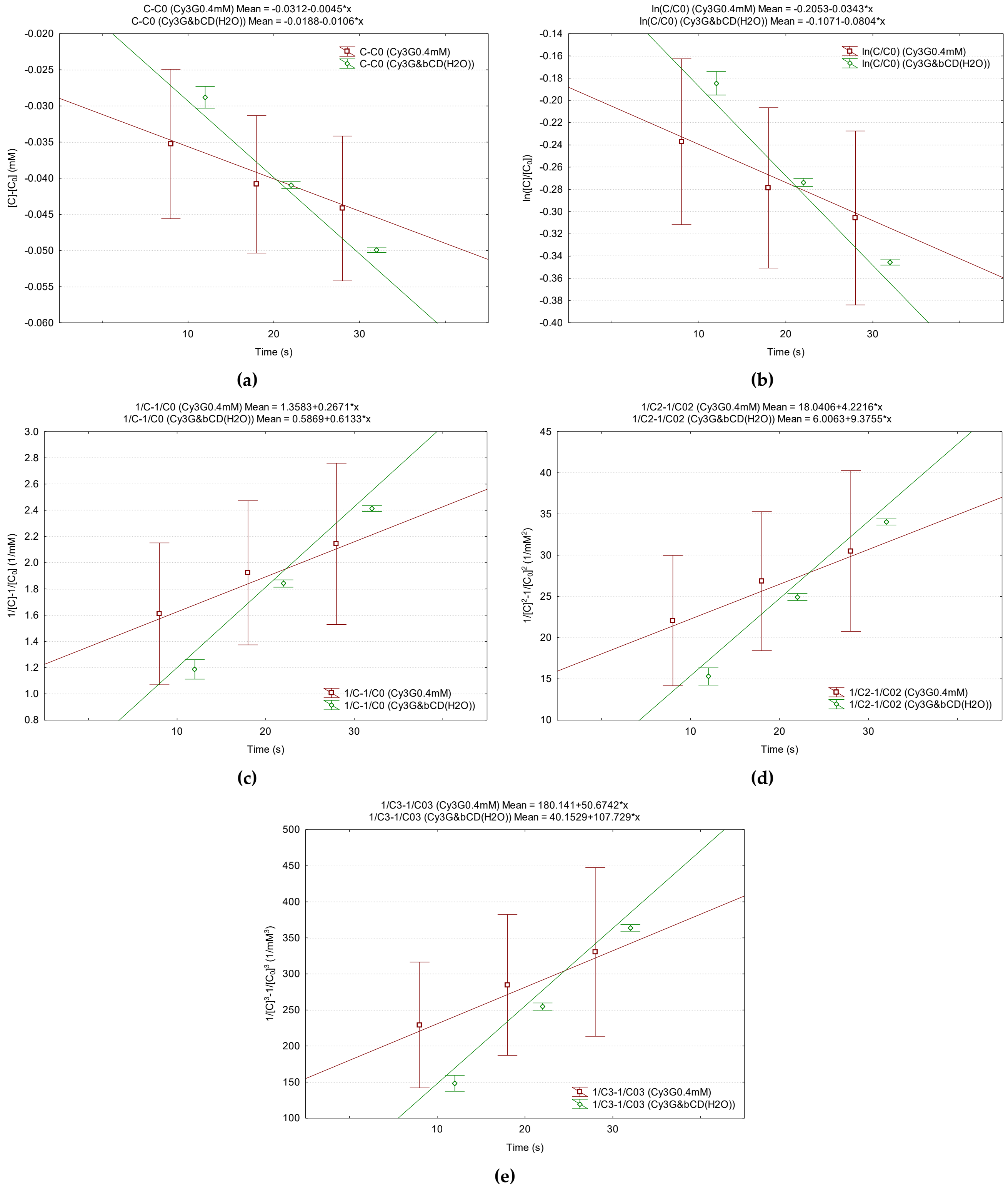

Figure S19. Integrated rate law representation for the zeroth–fourth-order models for the "fast" DPPH· reaction with the standard 0.4 mM cyanidin 3-*O*-glucoside (without or with β-cyclodextrin as additive during the spectrophotometric monitoring). For duplicate samples, Whisker type plots as mean ± 0.95·standard error were used.

Table S5. Kinetic and statistic parameters corresponding to the zeroth–fourth-order models for the "fast" DPPH· reaction with standard 0.4 mM cyanidin 3-*O*-glucoside (without or with β-cyclodextrin as additive during the spectrophotometric monitoring). On the same row, k values with different superscript capital letters are significantly different, according to Tukey's HSD test ($p < 0.05$). On the same row, $t_{1/2}$ values with different superscript lowercase letters are significantly different, according to Tukey's HSD test ($p < 0.05$).

**Parameter**	***Cy3G***	***Cy3G_bCD***
Rate constant, k_0 (mM/s)	0.0004 ^{A}	0.0011 ^{A}
Half-life, $t_{1/2(0)}$ (s)	213.8 ^{a}	77.7 ^{a}
Determination coefficient, $r^2_{(0)}$	0.980	0.993
Rate constant, k_1 (1/s)	0.0034 ^{A}	0.008 ^{B}
Half-life, $t_{1/2(1)}$ (s)	203.9 ^{a}	86.6 ^{b}
Determination coefficient, $r^2_{(1)}$	0.986	0.996
Rate constant, k_2 (1/(mM·s))	0.0267 ^{A}	0.0613 ^{B}
Half-life, $t_{1/2(2)}$ (s)	219.0 ^{a}	95.4 ^{b}
Determination coefficient, $r^2_{(2)}$	0.990	0.998
Rate constant, k_3 (1/(mM^2·s))	0.422 ^{A}	0.938 ^{B}
Half-life, $t_{1/2(3)}$ (s)	121.6 ^{a}	54.7 ^{b}
Determination coefficient, $r^2_{(3)}$	**0.994**	**0.9997**
Rate constant, k_4 (1/(mM^3·s))	5.067 ^{A}	10.77 ^{B}
Half-life, $t_{1/2(4)}$ (s)	92.1 ^{a}	43.3 ^{b}
Determination coefficient, $r^2_{(4)}$	**0.997**	**0.9999**

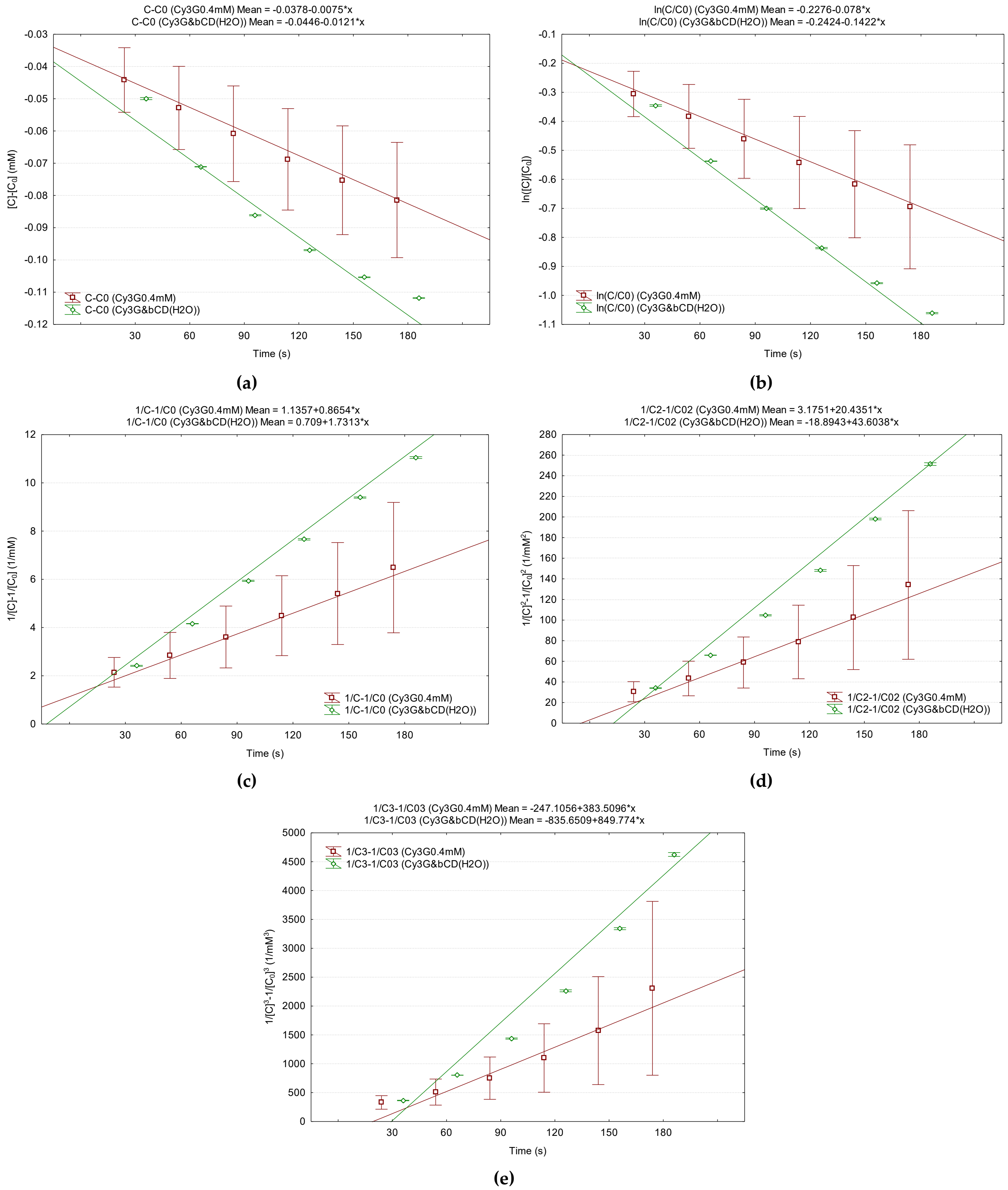

Figure S20. Integrated rate law representation for the zeroth–fourth-order models for the "slow" DPPH· reaction with the standard 0.4 mM cyanidin 3-*O*-glucoside (without or with β-cyclodextrin as additive during the spectrophotometric monitoring). For duplicate samples, Whisker type plots as mean ± 0.95·standard error were used.

Table S6. Kinetic and statistic parameters corresponding to the zeroth–fourth-order models for the "slow" DPPH· reaction with standard 0.4 mM cyanidin 3-*O*-glucoside (without or with β-cyclodextrin as additive during the spectrophotometric monitoring). On the same row, k values with different superscript capital letters are significantly different, according to Tukey's HSD test ($p < 0.05$). On the same row, $t_{1/2}$ values with different superscript lowercase letters are significantly different, according to Tukey's HSD test ($p < 0.15$).

Parameter	*Cy3G*	*Cy3G_bCD*
Rate constant, k_0 (mM/s)	0.0003^{A}	0.0004^{A}
Half-life, $t_{1/2(0)}$ (s)	285.0^{a}	213.8^{a}
Determination coefficient, $r^2_{(0)}$	0.996	0.965
Rate constant, k_1 (1/s)	0.0026^{A}	0.0048^{B}
Half-life, $t_{1/2(1)}$ (s)	266.6^{a}	144.4^{b}
Determination coefficient, $r^2_{(1)}$	**0.9999**	**0.990**
Rate constant, k_2 (1/(mM·s))	0.0287^{A}	0.0578^{B}
Half-life, $t_{1/2(2)}$ (s)	203.8^{a}	101.2^{b}
Determination coefficient, $r^2_{(2)}$	0.996	**0.9999**
Rate constant, k_3 (1/(mM²·s))	0.67^{A}	1.441^{B}
Half-life, $t_{1/2(3)}$ (s)	76.6^{a}	35.6^{a}
Determination coefficient, $r^2_{(3)}$	0.979	0.994
Rate constant, k_4 (1/(mM³·s))	12.42^{A}	27.76^{B}
Half-life, $t_{1/2(4)}$ (s)	37.6^{a}	16.8^{a}
Determination coefficient, $r^2_{(4)}$	0.948	0.973

11. Molecular modeling and computed properties for the main anthocyanins in berry extracts

Anthocyanin structures were built using the Model Build module from the HyperChem 7.52 software package (HyperCube, Inc., Gainesville, FL, USA). The most stable conformation of antioxidant structures were obtained using the MM+ Molecular Mechanics Force Field module and Polak-Ribiere (Conjugate gradient) in the Conformational Search module, considering all flexible bonds and rings (i.e., glucopyranoside moiety), up to 8 simultaneous variations, ranges of ±60 to 180° for acyclic torsion variation and ±30 to 120° for ring torsion flexing, usage directed searching method with an acceptance energy criterion of 6 kcal/mol above best, a root mean square (RMS) gradient of 0.01 kcal/(Å·mol) and a maximum cycles of 5000. The lowest energy conformation of the antioxidant compound was refined using the AM1 (Austin Model 1) semi-empirical method, with a total charge of +1 (attributed to the flavylium oxygen atom), spin multiplicity 3, Restricted Hartree-Fock (RHF) spin pairing. Then, the logarithm of the octanol/water partition coefficient, logP, was determined using the QSAR Properties program from the HyperChem package (logP calculation was based on the atomic parameters, according to Ghose et al. and Viswanadhan et al. [110-111]; "References" section in the main text).

Further, the total charge density in the range of -0.3 to 0.3 (mapped function range) was obtained for all anthocyanin structures in the most stable conformations. The total charge density contour value was set to 0.05, with a translucent and 3D mapped surface. The isosurface images for the most stable conformations of the cyanidin 3-*O*-glucoside (Cy3G), cyanidin (Cy) and pelargonidin 3-*O*-glucoside (Plg3G) are presented in Figures S21-S23, together with the range legend.

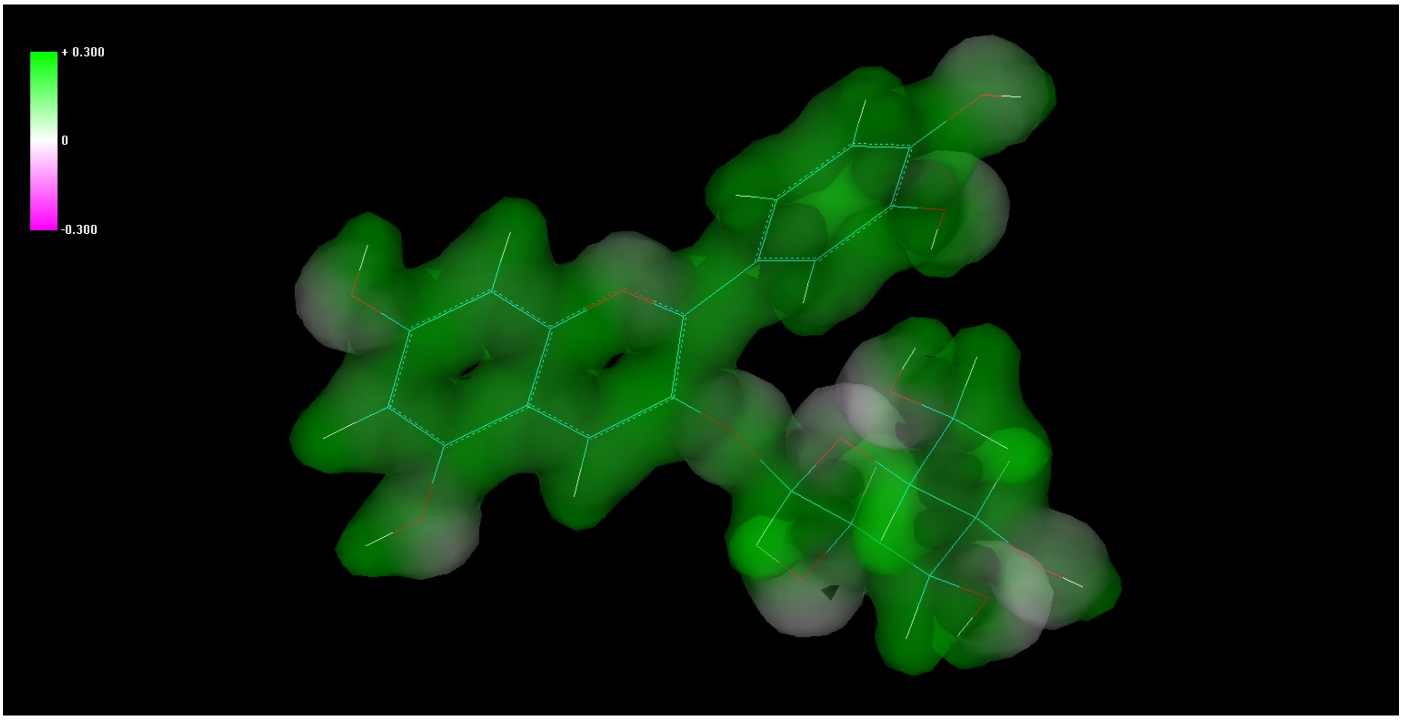

Figure S21. Isosurface image for the most stable conformation of the cyanidin 3-*O*-glucoside (Cy3G). The total charge density was in the range of -0.3 to 0.3 (mapped function range) and is shown in the upper-left corner as a range legend.

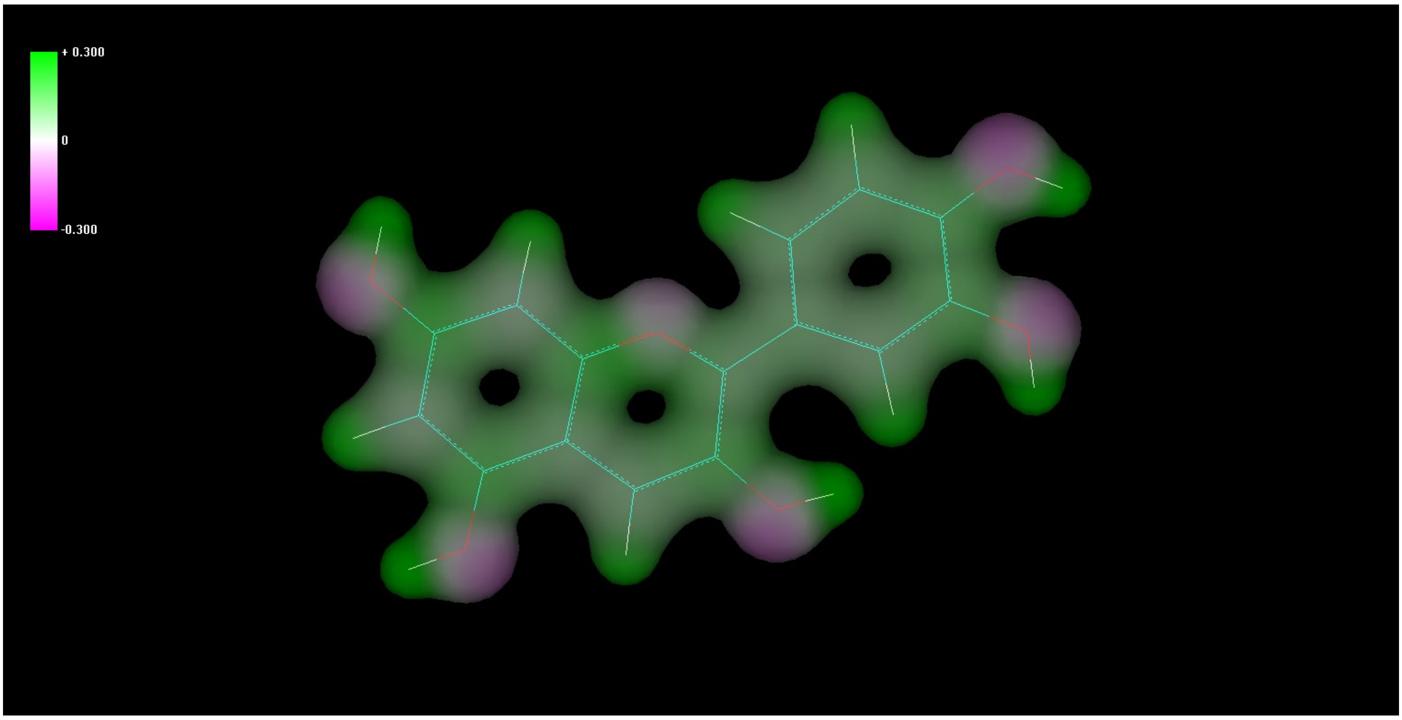

Figure S22. Isosurface image for the most stable conformation of the cyanidin (Cy). The total charge density was in the range of -0.3 to 0.3 (mapped function range) and is shown in the upper-left corner as a range legend.

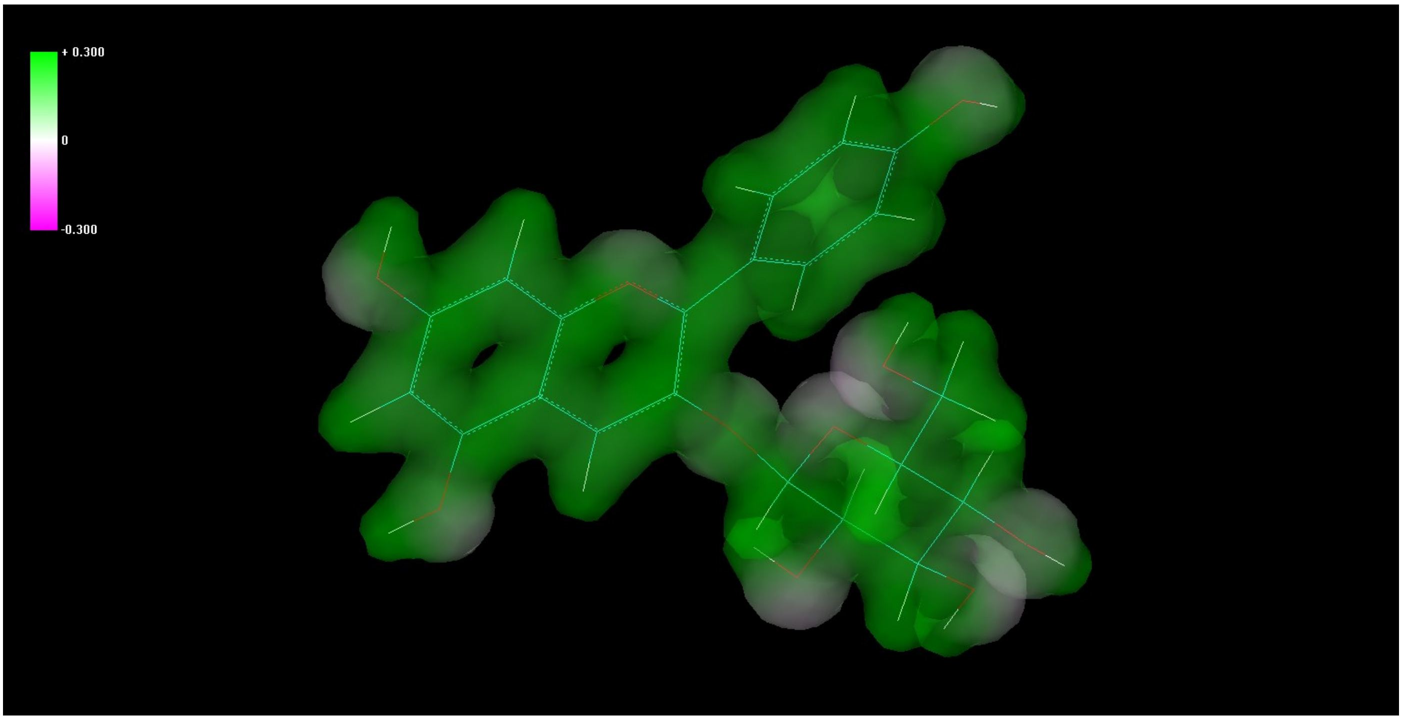

Figure S23. Isosurface image for the most stable conformation of the pelargonidin 3-*O*-glucoside (Plg3G). The total charge density was in the range of -0.3 to 0.3 (mapped function range) and is shown in the upper-left corner as a range legend.

12. Molecular modeling and docking of DPPH· and cyanidin 3-*O*-glucoside in β-cyclodextrin

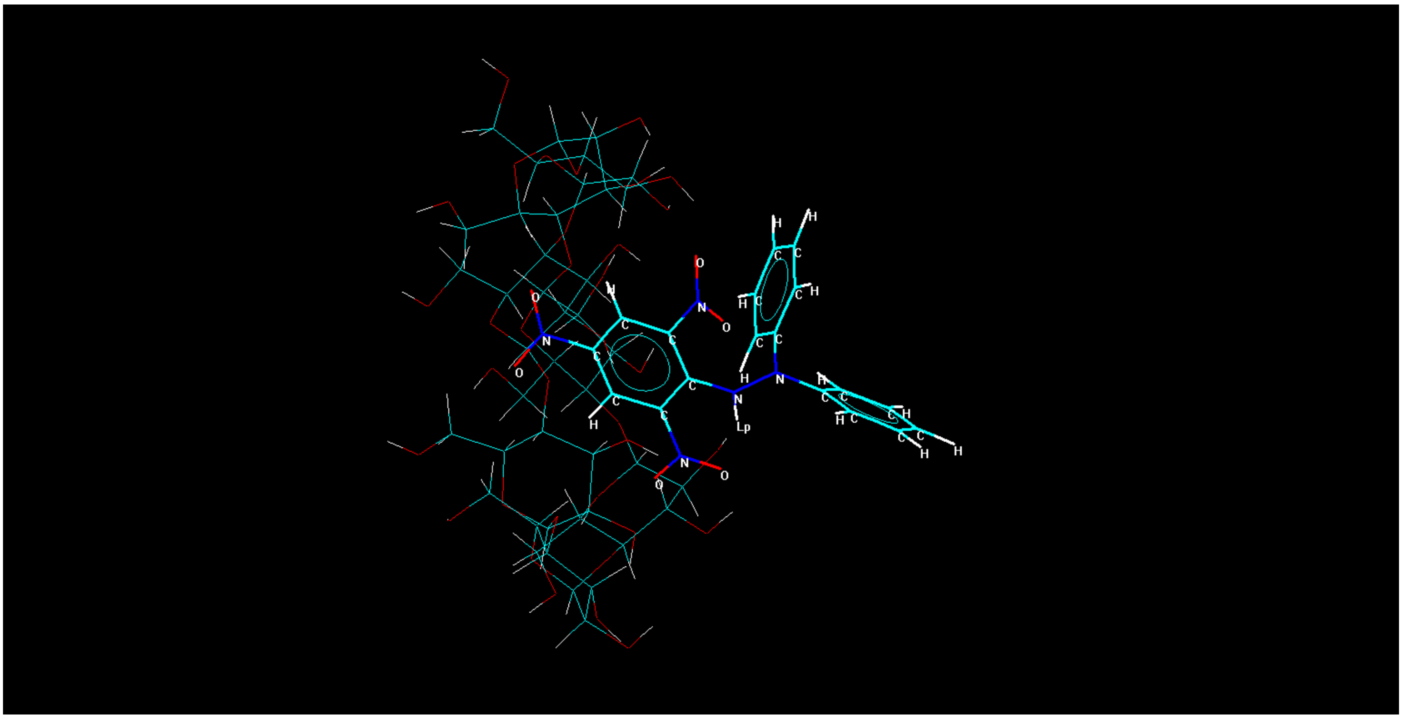

Figure S24. Optimized β-cyclodextrin / DPPH· complex, obtained with MM+ Molecular Mechanics Force Field module and Polak-Ribiere (Conjugate gradient) algorithm from the HyperChem 7.52 software package. Both β-CD and DPPH· were built and optimized separately as was presented above. The interaction starts with the DPPH· structure (both the Ph_2 and picryl sides) oriented to the secondary face of the β-CD, at a distance of ~8 Å between the gravity centers. DPPH· molecule is highlighted. The picryl moiety is only partial encapsulated into the β-CD cavity (at approximately 50%).

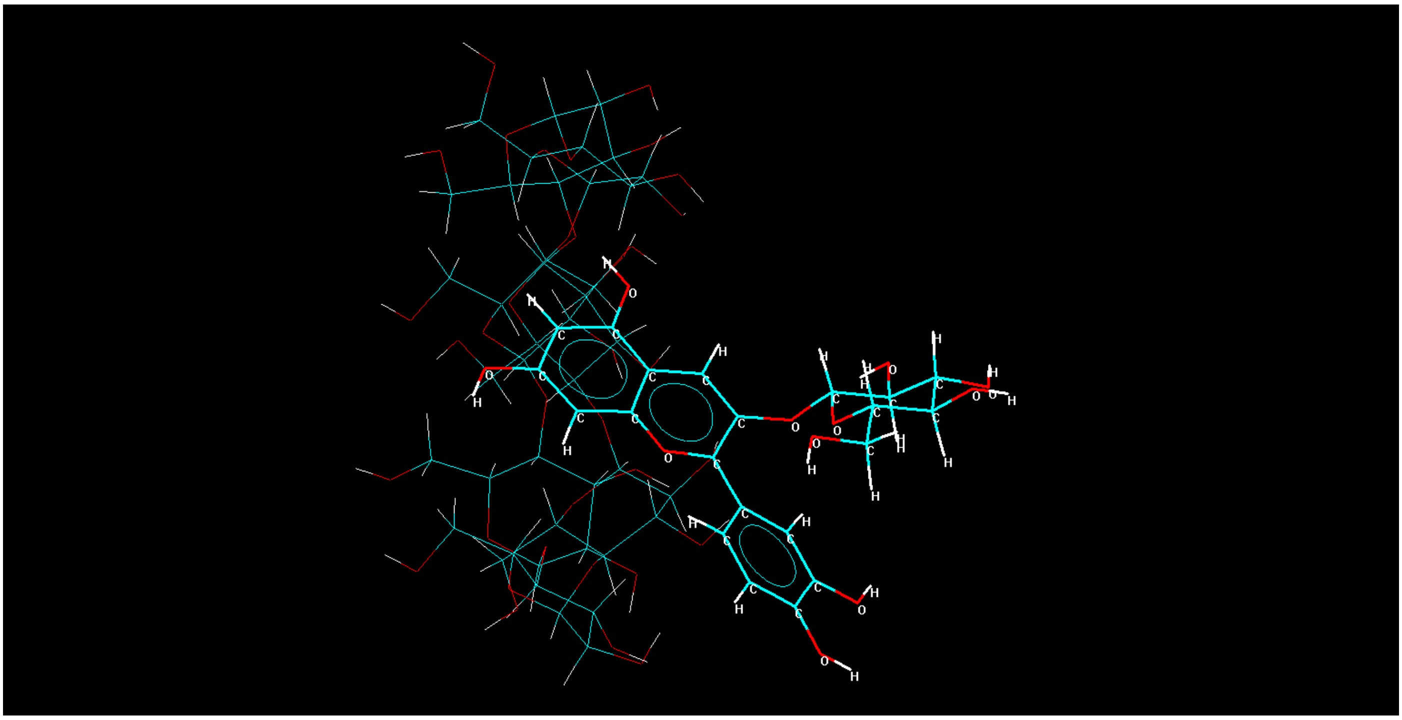

Figure S25. Optimized β-cyclodextrin / cyanidin 3-*O*-glucoside complex, obtained with MM+ Molecular Mechanics Force Field module and Polak-Ribiere (Conjugate gradient) algorithm from the HyperChem 7.52 software package. Both β-CD and Cy3G were built and optimized separately as was presented above. The interaction starts with the Cy3G structure (benzopyrane moiety) oriented to the secondary face of the β-CD, at a distance of ~8 Å between the gravity centers. Cy3G molecule is highlighted. Benzo moiety is completely encapsulated. The 2-phenyl moiety (not encapsulated) has the 3- and 4-OH groups oriented to the solution, as well as the glucose moiety. This favors the reaction with the DPPH· radical from the solution.
